# Supplementary material for: Distribution and diversity of ‘Tectomicrobia’, a deep-branching uncultivated bacterial lineage harboring rich producers of bioactive metabolites
Source: ISME Commun. 2023 May 29;3:50. doi: 10.1038/s43705-023-00259-z (PMC10227082; doi:10.1038/s43705-023-00259-z)
Supplement: Supplementary file 1 — Previous Manuscript File - For Editorial Use [file 43705_2023_259_MOESM1_ESM.docx]

**Distribution and diversity of 'Tectomicrobia', a deep-branching uncultivated bacterial lineage harboring rich producers of bioactive metabolites**

Eike E. Peters^1*^, Jackson K. B. Cahn^1*^, Alessandro Lotti^1*^, Asimenia Gavriilidou^2^, Ursula A. E. Steffens^3^, Catarina Loureiro^2^, Michelle A Schorn^2^, Paco Cárdenas^4^, Nilani Vickneswaran^3^, Phillip Crews^5^, Detmer Sipkema^2^, Jörn Piel^1†^

^1^Institute of Microbiology, Eidgenössische Technische Hochschule (ETH) Zürich, Vladimir-Prelog-Weg 4, 8093 Zürich, Switzerland.

^2^Laboratory of Microbiology, Wageningen University and Research, 6708 WE Wageningen, The Netherlands.

^3^Kekule Institute of Organic Chemistry and Biochemistry, University of Bonn, Gerhard-Domagk-Strasse 1, 53121 Bonn, Germany.

^4^Pharmacognosy, Department of Pharmaceutical Biosciences, BioMedical Center, Uppsala University, Husargatan 3, 75124, Uppsala, Sweden.

^5^Department of Chemistry and Biochemistry, University of California at Santa Cruz, Santa Cruz, California, 95064, United States.

^*^These authors contributed equally to the work

^†^Corresponding authors Email: [jpiel@micro.biol.ethz.ch](mailto:jpiel@micro.biol.ethz.ch)

Running title: Diversity of the candidate phylum ‘Tectomicrobia’

Subject Category: Microbial ecology and functional diversity of natural habitats

Conflict of Interest

The authors declare no competing financial interests.

**Abstract**

Genomic and functional analyses of bacterial sponge symbionts belonging to the uncultivated candidate genus ‘Entotheonella’ has revealed them as the prolific producers of bioactive compounds previously identified from their invertebrate hosts. These studies also suggested ‘Entotheonella’ as the first members of a new candidate phylum, 'Tectomicrobia'. Here we analyzed the phylogenetic structure and environmental distribution of this as-yet sparsely populated phylum-like lineage. The data show that 'Entotheonella' and other 'Tectomicrobia' are not restricted to marine habitats but widely distributed among terrestrial locations. The inferred phylogenetic trees suggest several intra-phylum lineages with diverse lifestyles. Of these, the previously described 'Entotheonella' lineage can be more accurately divided into at least three different candidate genera with the terrestrial *'Candidatus* Prasianella', the largely terrestrial 'Candidatus Allonella', the '*Candidatus* Thalassonella' comprising sponge-associated members, and the more widely distributed *'Candidatus* Entotheonella'. Genomic characterization of 'Thalassonella' members from a range of sponge hosts did not suggest a role as providers of natural products, despite high genomic similarity to ‘Entotheonella’ regarding primary metabolism and implied lifestyle. In contrast, the analysis revealed a correlation between the revised 'Entotheonella' 16S rRNA gene phylogeny and a specific association with sponges and their natural products. This feature might serve as a discovery method to accelerate the identification of new chemically rich 'Entotheonella' variants, and led to the identification of the first 'Entotheonella' symbiont in a non-tetractinellid sponge, *Psammocinia* sp., indicating a wide host distribution of 'Entotheonella'-based chemical symbiosis.

**Introduction**

The bacterial tree of life contains numerous deep-branching lineages that lack cultivated representatives [1-5]. Data from 16S rRNA genes and whole environmental genomes, obtained by metagenomic binning or single-cell sequencing, support the existence of dozens of uncultivated phylum-like divisions that are distributed ubiquitously or in more specialized habitats. Among a large and growing list of examples are the Candidate Phyla Radiation (CPR, previously known as 'Patescibacteria') [3, 6-8], the SAR324 group detected in hydrothermal plumes [4, 9], or *'*Poribacteria' present in sponge microbiomes [10]. 'Omics' data suggest diverse and intriguing functions for such elusive taxa [11, 12], but as experimental validation is usually challenging, verified functions remain limited.

In collaborative studies, we previously reported members of the candidate genus 'Entotheonella', first described by researchers at the Scripps Institution of Oceanography [13, 14], as an uncultivated taxon with a remarkably rich specialized metabolism [15-18]. Genome data suggested 'Entotheonella' as members of a new candidate phylum, termed 'Tectomicrobia' [15], which is corroborated by a more recent reanalysis of the bacterial tree of life based on standardized classification criteria [3]. All sequenced 'Entotheonella' phylotypes with known morphology form multicellular filaments, have large genomes around 10 Mb, and colonize sponges with which they appear to form mutualistic associations involving chemical defense [15-19] and arsenic and heavy metal detoxification [12]. Marine sponges are prolific sources of bioactive natural products that may contribute to protecting the sessile animals against predators and epibionts [20]. In the demosponge *Theonella swinhoei* (order Tetractinellida, suborder Astrophorina, family Theonellidae), containing a particularly rich chemistry, bioinformatic and biochemical data attributed most of the known substances to the symbiont phylotypes '*Ca.* Entotheonella factor' and '*Ca.* Entotheonella serta' [15, 16] (Table 1). These producers generate distinct sets of natural products and, in a mutually exclusive fashion, colonize two different host chemotypes, *T. swinhoei* Y and W, as members of microbiomes comprising numerous other bacteria [21, 22, 23], including the aurantoside producer '*Ca.* Poriflexus aureus' (Chloroflexi) present in the Y chemotype [24]. In this sponge, 'E. factor' is also accompanied by another closely related variant, 'Ca. Entotheonella gemina' [15], while a further 'Entotheonella' phylotype is present in a *T. swinhoei* W sampled from the Red Sea. One or more additional 'Entotheonella' phylotypes were detected in the theonellid sponge *Discodermia* *calyx* and collectively linked to the production of three biosynthetically distinct compound families [19, 25, 26].

Besides biosynthetic gene clusters (BGCs) assigned to known sponge natural products, all 'Entotheonella' genomes sequenced to date ('E. serta', 'E. factor', and 'E. gemina') contain multiple biosynthetic loci for as-yet unknown compounds. Enzymatic studies on two of the unassigned BGCs suggest that the orphan clusters are likely functional and encode the biosynthesis of previously unknown metabolites [15, 27, 28]. Their remarkable metabolic capabilities classify 'Entotheonella' as the first uncultivated producer taxon with a chemical richness and variability comparable to important culturable drug discovery sources, such as filamentous *Actinomycetota*, Cyanobacteria, or Myxobacteria [29]. Their pharmacological potential, intriguing biology, and isolated phylogenetic position warrant further investigations into the distribution and functions of 'Tectomicrobia', which currently contain only four members in the Genome Taxonomy Database [3].

In this study, we sought to obtain insights into the phylogenetic structure and environmental distribution of 'Tectomicrobia', seeking to address the following three questions: Do further tectomicrobial taxa exist besides 'Entotheonella'? If so, what is their chemical potential? Is the association with sponges a general feature of this lineage or can free-living representatives be identified? Here, we present new sequence data, which along with re-analysis of publicly available sequences and corroborated by recent studies [30-33], show that 'Tectomicrobia' contains a much wider range of lineages that occur in diverse marine and terrestrial habitats. For one of these, the new sponge-associated candidate genus 'Thalassonella', we present eight metagenome-assembled genomes (MAGs). Within 'Entotheonella', we identified two distinct phylogenetic signatures that suggest the existence of generalist sponge colonizers as well as host-specific members, the latter including all known natural product-rich phylotypes. Based on these data, we tested whether a 16S rRNA gene-based prioritization strategy can pinpoint new 'Entotheonella' producers. This strategy resulted in the discovery of a new 'Entotheonella' variant in the dictyoceratid sponge *Psammocinia* sp., showing that small molecule-based symbiosis within 'Entotheonella' is not restricted to the demosponge order Tetractinellida.

**Materials and Methods**

*Sponge collection and sequencing*

Specimens of *Psammocinia* sp. (Demospongiae, Dictyoceratida, Irciniidae) were collected by SCUBA diving at Milne Bay, Papua New Guinea (Zoological Museum of Amsterdam collection number ZMAPOR 19842 = UCSC coll. no. 03526), 12-18 m depth, in December 2003 at 9°37.214‘ S 150°57.332‘ E, 9°14.008‘ S 150°46.782‘ E, 9°14.147‘ S 150°47.173‘ E, and 9°19.868‘ S 150°43.906‘ E as described in [34] and preserved in 95% EtOH. Shallow water *Geodia barretti* (Demospongiae, Tetractinellida, Geodiidae) were collected by SCUBA diving in 2012 in the Bjørnsund Islands and Lysefjord, western Norway [35]. Other North Atlantic deep sea *Geodia* species (*G. atlantica, G. barretti, G. hentscheli, G. macandrewii, G. parva, G. pachydermata, G. phlegraei*) [36] were collected between 2004 and 2016 using remotely operating vehicles (ROVs) and trawls/dredges during various cruises in western Norwegian fjords, Kosterfjord (Sweden), Rockall Bank, the Greenland Sea, the Davis Strait, the Flemish Cap, Svalbard and the Galicia Bank. All specimens were preserved in 95% EtOH shortly after collection. DNA was extracted using the DNeasy Blood and Tissue Kit (Qiagen). Partial 16S rRNA genes were amplified by a nested PCR approach using the bacterial primers 27F (5'-AGA GTT TGA TCM TGG CTC AG-3') and 1492R (5'-TAC GGY TAC CTT GTT ACG ACT T-3') in a first round of PCR and the 'Entotheonella'-specific primers Ento238F (5'-CCG GTC TGA GAT GAG CTT GC-3') [14] and Ento1442R (5'-TCA CCC CAA TCA CCC CGC-3') [15] in the second round of PCR. The resulting DNA fragments were either sequenced directly or subcloned into pJet1.2 using the CloneJet PCR Cloning Kit (Thermo Scientific) prior to Sanger sequencing. Specimens of *G. barretti* (Gb1-Gb2, Gb4-Gb10) and *G*. *atlantica* (Ga3) for metagenomic sequencing were collected by dredging onboard R/V *Hans Brattström* of the University of Bergen from Korsfjord, south of Bergen, Norway (60°8.13’ N, 5°6.7’ E) in September and October 2017 (Table S1). They were chopped and flash frozen with liquid nitrogen upon collection and stored at -80 °C. Specimens (Gb126, Gb278 and Gb305) of *G. barretti* were collected during benthic trawls by the crews of the R/V *Pâmiut* of the Greenland Institute of Natural Resources during cruises conducted by Fisheries and Oceans Canada in the Davis Strait taken during the same season (Sept-Oct) from 2011–2015 and were stored at -20 °C (Table S1) [37]. All *G. barretti* samples were crushed in liquid nitrogen to a fine powder with pestle and mortar. Two hundred mg of sponge tissue material were disrupted by bead beating using milling balls (5x2 mm + 2x5 mm) and 2 steps of shaking for 20 s at 4,000 rpm as described in [38]. Tissue lysate was further used for DNA extraction with the AllPrep DNA/RNA/Protein Mini Kit (Qiagen) and sequenced by Novogene (Hong Kong) using the Illumina HiSeq PE150 platform.

Four sponges (*Fascaplysinopsis* sp. DOM10, *Geodia* sp. DOM14B, *Aplysina* sp. DOM33, *Desmacella* sp. DOM40) were collected by submersible near Portsmouth, Dominica (Table S1). Sponges were stored in RNALater (Thermo Fisher) at -20 °C until further processing. Sponge pieces were defrosted, rinsed with sterile artificial seawater, chopped into small pieces, added to a PowerBead Tube and subjected to shaking three times for 60 seconds at 6,000 x g using the Precellys® 24 Homogenizer (Bertin GmbH). DNA was subsequently extracted following the standard protocol of the DNeasy® PowerSoil® Pro Kit (Qiagen). Metagenomic DNA samples were sent to Novogene Europe (United Kingdom) for sequencing using the Illumina Novaseq6000 platform with the PE150 library and sequencing kits.

*Soil sampling and sequencing*

Soil samples were collected from 19 locations in Germany and Norway (Table S2). To isolate the metagenomic DNA of the samples, the PowerSoil® DNA Isolation Kit (MO BIO Laboratories, Carlsbad, CA, USA) was used. Partial 16S rRNA genes were amplified by the same nested PCR approach detailed above.

*Enrichment of soil bacteria*

The collected soil was resuspended in 0.9% (w/v) aqueous NaCl solution using a hand blender. The suspension was stirred with a magnetic stirrer for 20 min then left undisturbed for additional 10 min to allow for settling of particles. The supernatant was decanted through a 32 µm Nytex mesh and centrifuged at 100 × g, 1 000 × g and 4500 × g to separate the bacteria by their different sedimentation characteristics. Each bacterial fraction was resuspended in 0.9% (w/v) aqueous NaCl. For further separation and to remove remaining soil particles, density gradient centrifugation was employed: A Nycodenz cushion (60% (w/v) SERVA, Heidelberg) was placed beneath the bacterial suspension and centrifuged at 10 000 × g for 20 min at 4 °C. The cell interlayer was transferred to a new tube, washed with phosphate-buffered saline (PBS) and resuspended in 0.9% NaCl. For subsequent CARD-FISH studies, the enriched cells were either fixed in 4% paraformaldehyde (PFA, in PBS) or 100% EtOH at 4 °C overnight and subsequently stored at -20 °C until use.

*Isolation of filamentous bacteria from the sponge* Psammocinia *sp.*

Filament-enriched bacterial cell fractions from *Psammocinia* sp. were prepared by a modified protocol previously described for *Theonella* sponges [15]. Briefly, the sponge tissue was cut into small pieces, immersed in buffered calcium- and magnesium-free artificial sea water (CMF-ASW: 10 mM Tris-Cl, 449 mM NaCl, 9 mM KCl, 33 mM Na_2_SO_4_, 2 mM NaHCO_3_) and further processed using a mortar and pestle. The homogenized suspension was subsequently transferred to a Falcon tube and incubated with a mixture of collagenase I and IV (end concentration: 240 µg/ml) for 30 min at 37 °C. After 10-fold dilution with CMF-ASW and addition of 2.5 mM ethylene glycol tetraacetic acid (EGTA), the solution was incubated on a rotating wheel at 4 °C overnight. Next day, the solution was passed through a 32 µm Nytex mesh and centrifuged at 100 ×g for 10 min to pellet filamentous bacteria. The pellet was washed 3 times with 500 µl CMF-ASW and stored at 4 °C until use.

*Phylogenetic analysis*

Previously deposited tectomicrobial 16S rRNA gene sequences were retrieved from the GenBank sequence database by BLASTing the full-length 16S rRNA gene sequence of 'Entotheonella factor' (KF926817). Hits above a threshold of 75% identity (>1000 sequences) were aligned using MUSCLE [39], followed by manual alignment correction. A total of 811 sequences were selected to create an alignment with maximal sequence length and without gaps in the region corresponding to positions 337 to 1078 in the *E. coli* homolog. To the dataset were added 187 sequences belonging to known species of *Nitrospirota*, *Deltaproteobacteria* (now reclassified into three phyla, *Desulfobacterota*, *Myxococcota* and *SAR324*), *Alphaprotebacteria*, *Rokubacteria*, *Acidobacteriota* and *Nitrospinota* as outgroups. After a first round of phylogenetic analysis using the Neighbor-Joining method [40], the putative tectomicrobial dataset was reduced from 811 to 447 sequences, with length ranging between 700 and 745 bp, that fulfilled the monophyly criterion. The initial phylogram was generated by the Neighbor-Joining method with 500 bootstrap resamplings using the Jukes-Cantor method to compute evolutionary distances. To further test for a placement within ‘Tectomicrobia’, the phylogeny was also reconstructed with the Maximum Likelihood algorithm with 500 bootstrap resamplings using the Generalized Time Reversible model [40, 41] as suggested as the best substitution model by MEGA6. A discrete gamma distribution of 0.47 was used to model evolutionary rate differences among sites. The percentage of replicate trees in which the associated taxa clustered together in a bootstrap test of both algorithms are indicated in the final tree when either value was above 50%. All evolutionary analyses were conducted in MEGA6 [42] and visualized using the online tool iTOL [43]. A global identity matrix was used to calculate the median sequence identities (Hodges-Lehmann median) of all taxa, and a normal approximation was the basis for the upper and lower bounds for the 95% confidence interval.

*Metagenome assembly, binning, and bin classification*

Adapter removal, quality filtering and normalization was done using the BBtools suite v37.64 [44] with parameters ktrim=r k=23 mink=7 hdist=1 tpe tbo qtrim=rl trimq=20 ftm=5 maq=20 minlen=50. *G. barretti* and *G. atlantica* reads were normalized for coverage with parameters target=200 min=3. Filtered reads were assembled with metaSPAdes 3.12 [45] using the --meta and --only-assembler flags, and contigs were binned using metaWRAP v1.2 [46] with minimum completeness of 50%, maximum contamination of 10% (*G. barretti* samples were binned with MaxBin2 and metaBAT2 and “DOM” samples with MaxBin2, metaBAT2, and CONCOCT). The obtained bins were taxonomically classified using the GTDB-Tool Kit 1.1.0 (GTDB-Tk) *classify* workflow [47].

A BLASTN search using the 16S rRNA gene sequences from 'E. factor' and 'E. serta' as queries was used to detect 16S rRNA gene sequences within the putative 'Tectomicrobia' bins. Of the 24 putative 'Tectomicrobia' bins, 22 had at least a fragmentary 16S rRNA gene sequence, and 14 of those had at least 400 bp of overlap with the amplicon generated from our ‘Entotheonella’-specific primers [15, 16]. These sequences were aligned to the 811 used previously using MUSCLE, followed by trimming of the alignment to correct for different sequence lengths and manual realignment. The phylogenetic tree was regenerated for analysis using the Maximum Likelihood method as described above.

*Phylogenomic and genomic analysis*

Phylogenomic trees were constructed in Anvi'o [48] using the standard phylogenomics workflow laid out in the tutorial. Briefly, the putative 'Tectomicrobia' MAGs were imported into Anvi'o, genes were called with Prodigal [49], and an HMM profile was created. The protein sequences corresponding to 71 bacterial single-copy genes [50] were extracted, filtered to remove those present in less than 75% of MAGs, and concatenated; these concatenated sequences were then aligned with MUSCLE [39]. The alignment was first adjusted by manual trimming before being used to generate a phylogenomic tree using FastTree v2.1.11 [51] (Fig. S8). BGCs were predicted using bacterial AntiSMASH 5.0 with 'relaxed' detection strictness [52]. The quality of the ‘Thalassonella’ MAGs was estimated with CheckM v1.1.2 [53]. Only medium-high-quality MAGs (>75% completeness, <5% contamination) were considered for downstream analysis. Genome annotation was performed with Prokka v1.14.6 [54]. Assignment of Kyoto Encyclopedia of Genes and Genomes (KEGG) Orthologs (KO) and metabolic pathway reconstruction were conducted using the KofamKOALA [55] and BlastKOALA [56] online tools with default settings. For annotation with BlastKOALA, the ‘species_prokaryotes’ KEGG GENES database was used.

*Catalyzed reporter deposition fluorescence* in situ *hybridization (CARD-FISH)*

CARD-FISH experiments were performed as described previously [19]. Briefly, *Psammocinia* sp. was flash-frozen in liquid nitrogen and subsequently cut into 30 µm slices using a microtome (Microm, Thermo Fisher). Representative sponge tissue slices were then transferred onto microscopy slides, air-dried for 3 h at room temperature and subsequently fixed in PBS-buffered 4% PFA (4 °C, overnight). After washing with PBS, slices were permeabilized with 10 mg/ml lysozyme (20 mM Tris HCl pH 8.0, 2 mM EDTA, 0.1% Triton X-100) for 30 min at room temperature, washed once with PBS and incubated for 10 min at 4 °C with 1 mg/ml proteinase K (100 mM Tris HCl pH 8.0, 100 mM EDTA, 0.1% Triton X-100). Endogenous peroxidases were subsequently quenched by incubation with 0.01 M HCl for 20 min at room temperature, afterwards washed with PBS, dehydrated in pure ethanol for 3 min at room temperature, and air-dried. The hybridization reaction took place in hybridization buffer [0.9 M NaCl, 20 mM Tris·HCl pH 7.6, 10% (wt/vol) dextran sulfate, 0.05% SDS, 1% nucleic acid blocking reagent (Roche), 0.5 mg/mL herring sperm DNA (Sigma)] containing 55% (vol/vol) formamide and 0.5 ng/μL 'Entotheonella'-specific horseradish peroxidase (HRP)-coupled probe ESP219 (5′-CCG CAA GCY CAT CTC AGA CC-3′; BioMers) for 4 h at 35 °C. Subsequently, tissue slices were washed once in prewarmed washing buffer (3 mM NaCl, 5 mM EDTA pH 8.0, 20 mM Tris·HCl pH 7.6, 0.05% SDS) and once in PBS-T (0.01% Triton X-100) for each 30 min at 37 °C. After equilibration of the probe-delivered HRP in PBS for 20 min, tissue slices were incubated in amplification solution [1× PBS pH 7.6, 2 M NaCl, 20% (wt/vol) dextran sulfate, 0.1% nucleic acid blocking reagent (Roche), 0.0015% H_2_O_2_] containing Alexa Fluor 647-labeled tyramide (Life Science) for 1 h at 37 °C in the dark. Next, tissue slices were washed 3 times in PBS for 10 min at room temperature, once washed in distilled ice-cold H_2_O, dehydrated in pure ethanol, and air-dried. For microscopic analysis, tissue slices were covered with mountant (Citifluor Ltd) and observed under a Zeiss Axioskop 2 epifluorescence microscope equipped with a 75-W xenon arc lamp (XBO 75) and a 20× Plan Neofluar objective.

**Results and Discussion**

**Analysis of environmental samples for the presence of 'Tectomicrobia'**

Although our previous analyses had focused on 'Tectomicrobia' living in symbiosis with astrophorin sponges, preliminary phylogenetic analyses also suggested the existence of this candidate phylum in other sponges and habitats. In an attempt to uncover further tectomicrobial diversity, we engaged in a targeted sequence prospecting campaign. For this purpose, we collected soil from 19 locations in Germany and Norway (Table S2) and isolated the DNA of enriched bacterial fractions prepared from these samples. To analyze phylogenetic divergence in these samples we generated 16S rRNA gene sequences by a nested PCR approach previously established for the identification of 'Entotheonella' in metagenomic DNA samples [15] or by the use of the more general 16S rRNA gene PCR primers, 27F and 1492R, on enriched bacterial cell pellets. In both cases, target amplicons were obtained and subcloned for sequencing, resulting in a combined 45 new near full length tectomicrobial 16S rRNA gene sequences. A similar procedure was carried out on *Geodia* sponges collected from the North Atlantic as well as previously collected theonellid sponges from Japan and Israel [15, 18], generating an additional 35 new tectomicrobial 16S rRNA sequences, for a total of 80 (Table S3).

**Phylogenetic analyses suggest various marine and terrestrial genus-like lineages in ‘Tectomicrobia’**

For the phylogenetic analyses, public databases were searched for additional potential tectomicrobial 16S rRNA gene sequences to further enrich the dataset. In total, 447 sequences remained that were used in this study, consisting of 363 sequences derived from GenBank and our 84 newly acquired sequences. None of these originated from cultivated bacteria. A previous preliminary phylogenetic analysis of ‘Tectomicrobia’ had suggested the presence of three distinct clades, with the 'Entotheonella' lineage harboring most of the sequences [15]. Using our new, expanded dataset, phylograms inferred by maximum likelihood and neighbor-joining methods instead showed two deep-branching clades with high bootstrap support (Figs. 1A and S1-S3). Clade 1 comprised 119 sequences with low sequence divergence (94.3% median sequence identity, MSI) (Fig. 1B), suggesting a single candidate genus, for which we propose the name *'Candidatus* Allonella' (from Greek *ἄλλος*, other). Clade 2 was further divided into at least three major subgroups that were well-supported by bootstrap replicates. All sequences previously designated 'Entotheonella' [15] fell into one of these three subgroups, along with 11 of the sequences generated in our sequencing campaign. Subgroup 1, which retains the name ‘Entotheonella', had an MSI of 94.7%, suggesting a taxonomic ranking of a candidate genus according to the taxonomic thresholds proposed by Yarza *et al*. [57]. Subgroup 2 for which we propose the name *'Candidatus* Thalassonella' (Greek: *θάλασσα*, sea; MSI = 96.6%), contained 66 marine sponge-derived sequences, 52 of which were obtained from our sampling of deep-sea *Geodia* spp. The third genus-like subgroup in Clade 2 emerged with the addition of the new soil-derived 16S rRNA sequences generated in this study, and contained 112 sequences of exclusively terrestrial origin. Because the first soil sample that was positively tested for Tectomicrobia originated from a vegetable garden, we propose the name '*Candidatus* Prasianella' (ancient Greek, *πρασιά*, garden bed; MSI = 95.3%). In addition to their distinctive phylogeny, 'Prasianella' and 'Thalassonella' 16S rRNA gene sequences contained as a diagnostic feature a 25-29 bp insertion in the V3 region, which 'Entotheonella' sequences lacked (Fig. S4). Two additional smaller subgroups, 't1' and 't2', which contained 10 and 11 sequences of mainly terrestrial origin respectively, were also present in Clade 2 (Fig. S2). However, further sequences are necessary to resolve the phylogenetic relationship between 't1', 't2' and members of 'Thalassonella' and 'Prasianella'. Comparison of the groups within Clade 2 showed 85.4-90.3% MSI (Fig. 1B), suggesting that Clade 2 represents a candidate family, 'Entotheonellaceae', whereas Clades 1 and 2 had an MSI of 80.8%, suggesting membership in a shared class or phylum according to Yarza *et al.* thresholds [57]. Although our previous efforts to identify reservoirs of 'Tectomicrobia' had focused on marine sponges, the current analysis shows that members are widespread in soil. In total, 54.1% of the tectomicrobial sequences derive from soil habitats, 5.0% from freshwater, 1.4% from saltwater and 39.5% from marine sponges (Fig. 1C), although this distribution is likely heavily affected by sampling bias.

**Relationship between phylogeny and environmental distribution within 'Tectomicrobia' lineages**

All 112 sequences within the genus 'Prasianella' originated from terrestrial sources, but no clear further differentiated relationship could be identified between phylogeny and geographic origin (Fig. S3). Similarly, the geographic distribution of 'Thalassonella' covered various marine regions including the Pacific, Atlantic, Arctic and Indian Oceans (Fig. 2). Among the 66 'Thalassonella' sequences, most originated from various *Geodia* spp. sponges (37 sequences), followed by the 16 sequences from the two *Xestospongia* species *Xestospongia testudinaria* (Manado, Indonesia) and *Xestospongia muta* (Key Largo, Florida, USA) [58]. The large majority of sponges with ‘Thalassonella’ are high-microbial abundance (HMA) sponges (*Geodia* spp., *Xestospongia* spp., *Aplysina* spp., *Ecionemia alata*, *Ircinia strobilina, Vaceletia crypta, Plakortis halichondrioides*), with one low-microbial abundance (LMA) sponge (*Tedania ignis*) and two species of more uncertain status (*Astrosclera willeyana,* *Haliclona tubifera*) [59-61]. ‘Thalassonella’ subclades showed a high frequency of shared sponge hosts (phylosymbiosis), particularly for the two sponge genera for which several 'Thalassonella' sequences were available, *Geodia* and *Xestospongia* (Fig. 2). The 'Thalassonella' group also contained a single sequence from a diseased coral (*Montastraea faveolata*) as the only non-sponge-derived representative. However, since a corresponding sequence was not reported from a nearby, healthy coral, it might originate from a contamination rather than the coral microbiome [62]. Although no geographic patterns were observed (Fig. 2), sequences from similar water depths clustered together, albeit with moderately high bootstrap support, as apparent from the existence of individual 'Thalassonella' *Geodia* subclades comprising sublittoral to upper bathyal (36-787 m) and lower bathyal members (688-1462 m), as well as a 'Thalassonella' *Xestospongia* subclade comprising sublittoral (36-200 m) members (Fig. 2). Most *Geodia* tested had only one ASV, which was confirmed by subcloning 12 specimens; the exceptions were *G. barretti* PC619 and PC738 from Norway, *G. barretti* PC958 from Davis Strait, and *G. phlegraei* PC12 from Norway, which had two ASVs (Table S3). Thus, water depth, or more likely water masses, along with the sponge host species, appeared to be major determinants influencing the distribution of 'Thalassonella'. The sharing of specific ASVs by different *Geodia* species over large geographical distances could reflect *Geodia* population connectivity [63]. For example, *G. barretti* and *G. macandrewii* sharing one ASV between the upper bathyal Svalbard and Davis Strait (~3700 km apart) may reflect host-microbe connections via the deep-sea East Greenland current, followed by the West Greenland current. Depth has been reported in several studies as a factor that stratifies marine bacterial communities at the species (e.g., *Bacillus cereus* [64]), phylum (e.g., SAR11 [65]), and global level [66]. However, more thorough sampling will be required to rule out potential confounding factors.

**'Entotheonella' members exhibit two contrasting phylogenetic patterns**

Phylogenetic insights were of particular interest for members of 'Entotheonella' as rich producers of bioactive natural products in sponges [15, 16, 18, 19, 25, 26]. In contrast to 'Thalassonella', only limited relationship was initially apparent between bacterial phylogeny and host affiliation (Fig. 3). Also, host sponges here were a mix of HMA (*Theonella, Discodermia, Aplysina, Ircinia, Agelas, Pseudoceratina purpurea*) and LMA (*Cliona,* *Stylissa, Hexadella, Dysidea, Callyspongia*) sponges [59, 67]. However, closer analysis revealed two distinct distribution patterns among 'Entotheonella' phylotypes. All sequences corresponding to the previously described metabolically talented 'Entotheonella' phylotypes fell into one clade (group I in Fig. 3, MSI = 96.3% in Fig. S5) with relatively high sequence divergence and a branching topology that suggested some degree of specificity with sponge hosts. A contrasting pattern was found in groups IIa (MSI = 98.5%) and IIb (MSI = 99.4%) (Fig. 3 and S5), which harboured 'Entotheonella' sequences with highly similar 16S rRNA genes that were detected exclusively by PCR in a wide range of sponges. These closely related 'Entotheonella' phylotypes are clearly not tied to the phylogeny of their hosts; they might either be host-promiscuous symbionts or non-symbiotic contaminants from seawater. In some sponge species, e.g., *T. swinhoei* or *Discodermia dissoluta*, the same sponge specimen was found to contain sequences from groups I and IIa/IIb. The final 'Entotheonella' clade (group IIc with low bootstrap support) showed slightly higher sequence divergence (MSI = 97.3%) than groups IIa and IIb. While full-length 16S rRNA gene sequences might be needed to improve bootstrap values, It is noteworthy that the ‘Entotheonella’ lineage contains so many different phylotypes, both in absolute terms and relative to the phylum as a whole. Given the large number of BGCs in each ‘Entotheonella’ genome studied to date and the uniqueness of each BGC inventory [16, 28], this suggests a wealth of biosynthetic novelty yet to be discovered.

Since group I included all biosynthetically talented ‘Entotheonella’ previously characterized by genomic, microscopic, and spectroscopic methods, we were intrigued by the presence of additional group I members from terrestrial habitats, including soil samples collected at two distant locations in Germany and samples derived from dump layers of the leafcutter ant *Atta colombica*. However, our attempts to use mechanical enrichment and CARD-FISH-based visualization methods [18] to further characterize 'Entotheonella' in positive soil samples were unsuccessful.

**A potential 'Entotheonella' source of the anticancer polyketide psymberin in the sponge *Psammocinia* sp.**

The data on group I containing all known symbiotic 'Entotheonella' variants raised the question of whether additional sponge-associated producers can be identified in this clade. A promising candidate was a sequence amplified from metagenomic DNA of the sponge *Psammocinia* sp. (order Dictyoceratida, family Irciniidae) with 96.1% identity to the 16S rRNA gene of 'Entotheonella factor'. This sponge was previously shown to contain psymberin [68, 69], a cytotoxin with nanomolar activity against a variety of tumor cell lines [69, 70]. Psymberin belongs to the pederin-type family of polyketides that are produced by symbionts from diverse bacterial phyla [71], including onnamides and theopederins in *T. swinhoei* by 'E. factor' [15] and mycalamides in the sponge *Mycale hentscheli* by a bacterium of the gammaproteobacterial UBA10353 group [72]. From a metagenomic DNA library of *Psammocinia* sp., we had previously isolated the BGC for psymberin, but the producer could not be identified at the time [73]. However, re-analysis of the genes surrounding the isolated gene cluster showed high amino acid sequence identity (81-99%) to 'Entotheonella' proteins for three gene products (Fig. S6B, Table S4), suggesting an 'Entotheonella' symbiont as the possible source of psymberin. Initial attempts to detect filamentous bacteria in *Psammocinia* sp. by the established mechanical enrichment technique were unsuccessful, but a modified protocol involving the addition of collagenases and EGTA released copious amounts of multicellular filaments from the sponge extracellular matrix. To further characterize these filaments, we performed catalyzed reporter deposition fluorescence in situ hybridization (CARD-FISH) experiments using the previously reported 'Entotheonella'-specific probe ESP-219 on 30 µm sponge tissue slices [18]. This method resulted in selective labeling of the filamentous bacteria (Fig. 4 and S6C), which we subsequently named *'Candidatus* Entotheonella consociata' for its tight association with the sponge matrix (Latin, *consociata*; associated). The localization of these filaments to the inner pore surfaces of the sponge resembles previous findings in *Theonella* sponges [17, 18]. *Psammocinia* representing the first reported non-astrophorin sponge genus containing 'Entotheonella' and even belonging to a different sponge subclass (Keratosa) underlines the importance of this taxon for the biology of sponges. Efforts to directly sequence DNA isolated from the enriched cell pellet were unsuccessful, a phenomenon that we have also encountered for other 'Entotheonella'-containing samples [16, 18] but that might be overcome by single-bacterial sequencing [74].

**Metagenome-assembled genomes of '*Ca.* Thalassonella' from diverse sponges lack biosynthetic richness**

The lack of genomic information about 'Tectomicrobia' other than 'Entotheonella' is a self-reinforcing problem. Due to the lack of reference genomes against which to compare draft metagenome-assembled genomes (MAGs), these newly sequenced MAGs cannot be unambiguously assigned to the candidate phylum, or to the subordinate taxonomic groups introduced in this study but defined from 16S rRNA gene relatedness. This can be a challenge for phylogenomic inference tools such as the GTDB [3], which assigns taxonomy based on genome similarity to this limited reference set. Several metagenomics papers have mentioned the presence of 'Tectomicrobia' in metagenomes without addressing how these sequences relate to the available 'Entotheonella' data [30-33]. Because of their conservation, 16S rRNA gene sequences are rarely assembled successfully from metagenomes, preventing a direct comparison of these 'Tectomicrobia' MAGs with the 16S rRNA gene-based phylogeny presented here.

Among the metagenomes of 17 sponges recently sequenced by our groups, the GTDB-Tk assigned 24 MAGs with completeness greater than 50% and no more than 5% contamination as belonging to 'Tectomicrobia' (Table S1). Crucially, BLAST searches found that 14 of these MAGs contained 16S rRNA gene sequences with lengths ranging from 399 to 747 bp. These sequences were aligned to those used for phylogenetic analysis in this study. Five of these MAGs clustered with reference groups of 'Tectomicrobia', highlighting the challenge of phylogenomic assignment in a sparsely covered region of sequence space, while one diverged early from 'Entotheonellaceae' but could not be placed into a candidate genus, and the remaining nine fell clearly into the candidate genus 'Thalassonella' (Fig. 5A and Table S1). Although the process of trimming the alignments to accommodate these incomplete 16S rRNA gene sequences removed some of the subgenus level resolution in the phylogenetic tree, the biogeographic signatures within 'Thalassonella' can still be distinguished in the condensed tree (Fig. S7), and the groups proposed in this study correctly distinguish *Geodia*-derived MAGs from those derived from non-astrophorin sponges.

Finally, a phylogenomic tree was constructed in Anvi'o using these 15 MAGs for which 16S rRNA gene sequences were available, together with, three 'Entotheonella' MAGs, and three MAGs from the phylum Nitrospinota as references (Fig. 5B). This tree, based on a concatenated alignment of 71 bacterial marker genes, shows remarkable concordance with the 16S rRNA gene-based tree, supporting all assignments at subgenus levels within the phylum 'Tectomicrobia'. The only exception is Gb4_35, which had an inconclusive ‘Entotheonellales’ designation based on its 16S rRNA gene, but which phylogenomics reveals to belong to the ‘sublittoral to upper bathyal *Geodia* group within ‘Thalassonella’. Incorporation of putative 'Tectomicrobia' bins lacking 16S rRNA gene sequences identified an additional seven MAGs as 'Thalassonella', and rejected two additional MAGs as being incorrectly assigned to 'Tectomicrobia' (Fig. S8 and Table S1), but uncovered no genomes belonging to other candidate 'Tectomicrobia' genera, either 'Entotheonella' or the yet-elusive 'Allonella' or 'Prasianella'.

To assess the biosynthetic potential of 'Thalassonella', all candidate 'Tectomicrobia' genomes were analyzed using antiSMASH. In contrast to the numerous and largely unique BGCs of 'Entotheonella' [15, 16, 28], the 'Thalassonella' MAGs possess just 2-5 BGCs each, nearly all of which are conserved within the genus (Fig. 6, S9-13). Of particular note are two putative terpene BGCs -- one present in all 17 'Thalassonella' MAGs and the other present in 11 of the 17 -- that are also found in the three published 'Entotheonella' draft genomes [15-17]. A putative BGC for a modified lipid is present in 15 of 17 'Thalassonella’ MAGs, and a unimodular type I polyketide synthase is found exclusively but universally in the 'sublittoral to upper bathyal *Geodia*' subgenus grouping. An orthologous PKS was previously observed in the sequenced metagenome of the sponge *Plakortis simplex* and six additional sponges, and given the designation *swf* [75], but those studies did not suggest a bacterial source. The reported *swf* gene clusters show clear sequence and organizational homology but are more divergent from the 'Thalassonella' sequences of this study than they are from one another (Figure S13). Neither the lipid-modifying cluster nor the *swf* locus are found in 'Entotheonella' genomes, and none of the four conserved 'Thalassonella' BGCs are found in the outgroup MAGs. Although the functions of these clusters are unknown, their hypothetical products could be useful molecular biomarkers for the presence of 'Tectomicrobia' generally, in the case of the terpenes, or 'Thalassonella' specifically, in the case of the other BGCs. Indeed, using the terpene synthase in sequence similarity searches retrieved two MAGs that became publicly available after the completion of this study and that were both classified as 'Tectomicrobia' members (Genbank accession VGLS01001192 and [76]).

**‘*Ca.* Thalassonella’ members lead a similar lifestyle to ‘Entotheonella’**

Functional characterization and metabolic pathway reconstruction of 8 medium-high-quality ‘Thalassonella’ MAGs revealed important clues about their primary metabolism. On average, the analyzed MAGs were 87% complete and 2% contaminated with a genome size of 3.6 Mbp and 63% GC content (Table S5). Comparative genomic analysis suggested that ‘Thalassonella’ has a facultative anaerobic, heterotrophic metabolism highly similar to that previously reported for ‘Entotheonella’ [17]. The presence of genes coding for components of the respiratory chain and for oxygen-tolerant enzymes indicate the capacity of ‘Thalassonella’ to use oxygen as the terminal electron acceptor. Various types of cytochrome c oxidases (low and high O_2_ affinity) identified in the genomes support previous findings on the ability of sponge symbionts to survive under different oxygen concentrations prevailing in actively pumping sponges [77, 78]. Similarly to ‘Entotheonella’, multiple genes encoding CoA-transferases of family III (e.g. formyl-CoA transferase, benzylsuccinate CoA-transferase) and putative pathways for pyruvate fermentation to acetoin and 2,3 butanediol hint at a facultative anaerobic metabolism [17]. ‘Thalassonella’ MAGs harbored an almost complete set of genes for glycolysis (Embden-Meyerhof-Parnas pathway), the tricarboxylic acid (TCA) cycle and the pentose phosphate pathway (non-oxidative phase) (Table S6). It should be noted that the incompleteness of certain metabolic pathways must be interpreted cautiously as it could be attributed to sequencing or binning artifacts. However, in some cases key enzymes involved in core metabolic pathways might have alternatives performing the same function. For example, ‘Thalassonella’ MAGs were missing one of the key enzymes of the TCA cycle, namely the NADP-dependent isocitrate dehydrogenase [EC: 1.1.1.42], which is found in most prokaryotes, including ‘Entotheonella’ [17]. Instead, they contained the eukaryotic-type NAD-dependent isocitrate dehydrogenase [EC:1.1.1.41]. No autotrophic fixation pathways were identified in any of the genomes. A heterotrophic lifestyle was also reflected in the presence of various ATP-binding cassette (ABC) transporters for amino acids, oligosaccharides, lipopolysaccharides, and lipoproteins (Table S6). Gene-based evidence suggests that ‘Thalassonella’ has the capacity of using both methanol and oxalate as energy sources following the same energy acquisition strategy as ‘Entotheonella’. Methanol is considered a key fuel for marine microorganisms sourced from atmospheric deposition [79]) and phytoplankton production [80]. In the case of oxalate, it has been often observed in marine aerosols [81] and in the form of calcium oxalate in sponges [79]. ‘Thalassonella’ was also predicted to produce several amino acids, cofactors, and vitamins (Table S6). This biosynthetic potential has been previously reported for several sponge symbiotic lineages [17, 33, 83]. Since sponges are not capable of synthesizing several of these compounds, they are thought to obtain them either via filter-feeding or from their associated microorganisms [84]. Our findings suggest that members of the candidate genus ‘Thalassonella’ carry many traits typically found in sponge symbionts. Further, ‘Thalassonella’ members appeared to follow a similar lifestyle to ‘Entotheonella’ which contradicts the large difference in their BGC potential.

**Conclusions**

In the present study, the phylogenetic analysis of 447 16S rRNA gene sequences, including marine and terrestrial samples obtained by targeted sequence prospecting, demonstrates that the candidate phylum 'Tectomicrobia' is composed of a diverse range of phylotypes globally distributed among various habitats. The phylum can be divided into several clades, some of which can be preliminarily associated with environmental factors such as, water depth, or association with marine sponges. In contrast to 'Entotheonella', the first draft genomes from the candidate genus 'Thalassonella' do not suggest natural product richness, while the primary metabolism of both genera appears to be highly similar. However, it remains unknown whether '*Ca*. Entotheonella' represents the only talented genus within ‘Tectomicrobia’ due to the lack of additional tectomicrobial genomes spanning the remaining clades.

The analysis of the 'Entotheonella' genus suggests the existence of a subgroup of 'Entotheonella' members in phylosymbiosis with sponge hosts and a larger group of bacterial phylotypes that are highly similar to each other and show no co-speciation with the host. All of the known bioactive natural product producers in 'Tectomicrobia' fall into the former group. This interconnection offers an interesting possibility for the prioritization of sponges harboring group I 'Entotheonella', and has already led to the discovery of the first potential chemically productive 'Entotheonella' species in a non-astrophorin sponge. The evidence suggests that this symbiont, from *Psammocinia* sp., is the producer of the cytotoxic compound psymberin. Surprisingly, our studies also point towards the existence of bacteria within the 'Entotheonella' superproducer group I that are not associated with marine sponges, but no identification was possible beyond the 16S rRNA gene amplicon as enrichment experiments were not met with success. Further studies will therefore be needed to evaluate whether they are as chemically productive as their sponge-associated counterparts and can be cultivated more easily.

**Data availability**

16S rRNA gene sequences are available from the NCBI Sequence Read Archive under accession number OL753566 - OL753644 and OM256467; 'Thalassonella' MAGs are available from the European Nucleotide Archive under accession number ERS12129423 - ERS12129445.

**Acknowledgements**

The authors wish to thank Roy Meoded and Eric Helfrich for support with 'Entotheonella' detection, Shirley Pomponi for *D. dissoluta* samples, Ellen Kenchington and Wojciech Walkusz for the Canadian *Geodia* samples, Hans Tore Rapp for his repeated help to sample the Norwegian fjord *G. barretti* specimens, and Adriaan Schrier for offering his submarine to collect sponges in Dominica. Other sponge collectors: Pilar Ríos (specimens from Spain), Magnus Tornes (shallow *G. barretti* in Norway), Alexander Plotkin (Svalbard). This research was financially supported by the Gordon and Betty Moore Foundation (#9204, DOI: https://doi.org/10.37807/GBMF9204), the Swiss National Science Foundation (205320_185077), and the ETH (ETH Research Grant to JP), the European Commission through Horizon2020 project SponGES (Grant agreement ID: 679849) to DS, PCa and AG, the Marie Sklodowska-Curie Individual Fellowship COSMos (Grant agreement ID: 897121) to MAS, and the NIH (Grant R01 CA 47135) to PCr. This document reflects only the authors’ view and the Executive Agency for Small and Medium-sized Enterprises (EASME) is not responsible for any use that may be made of the information it contains.

**Author contributions**

EEP and JC performed phylogenetic analysis. JC performed phylogenomic and genomic analysis. EEP, US, PCa, and NV generated 16S rRNA gene sequences, CL, AG, and MAS performed processing and binning the metagenomes and AG performed the metabolic reconstruction of 'Thalassonella' PCr, DS, AG, and PCa provided the sponge samples. All authors designed research. EEP, JC, AL, AG, and JP wrote the manuscript with the assistance of the other authors.

**Compliance with ethical standards**

**Conflict of interest** The authors declare no competing interest

**References**

1. Amann RI, Ludwig W, Schleifer KH. Phylogenetic identification and in situ detection of individual microbial cells without cultivation. Microbiol Rev. 1995;59:143-69.
2. Konstantinidis K, Rosselló-Móra R, Amann R. Uncultivated microbes in need of their own taxonomy. ISME J. 2017;11:2399-2406.
3. Parks DH, Chuvochina M, Waite DW, Rinke C, Skarshewski A, Chaumeil P-A, et al. A standardized bacterial taxonomy based on genome phylogeny substantially revises the tree of life. Nat Biotechnol. 2018;36(10):996-1004.
4. Parks DH, Chuvochina M, Chaumeil P-A, Rinke C, Mussig AJ, Hugenholtz P. A complete domain-to-species taxonomy for Bacteria and Archaea. Nat Biotechnol. 2020;38(9):1079-86.
5. Hugenholtz P, Chuvochina M, Oren A, Parks DH, Soo RM. Prokaryotic taxonomy and nomenclature in the age of big sequence data. ISME J. 2021;15:1879-1892.
6. Brown CT, Hug LA, Thomas BC, Sharon I, Castelle CJ, Singh A, et al. Unusual biology across a group comprising more than 15% of domain Bacteria. Nature. 2015;523(7559):208.
7. Hug LA, Baker BJ, Anantharaman K, Brown CT, Probst AJ, Castelle CJ, et al. A new view of the tree of life. Nat Microbiol. 2016;1:16048.
8. Rinke C, Schwientek P, Sczyrba A, Ivanova NN, Anderson IJ, Cheng J-F, et al. Insights into the phylogeny and coding potential of microbial dark matter. Nature. 2013;499:431.
9. Cao H, Dong C, Bougouffa S, Li J, Zhang W, Shao Z, et al. Delta-proteobacterial SAR324 group in hydrothermal plumes on the South Mid-Atlantic Ridge. Sci Rep. 2016;6(1):1-9.
10. Kamke J, Rinke C, Schwientek P, Mavromatis K, Ivanova N, Sczyrba A, et al. The candidate phylum Poribacteria by single-cell genomics: new insights into phylogeny, cell-compartmentation, eukaryote-like repeat proteins, and other genomic features. PLoS one. 2014;9(1):e87353.
11. Crits-Christoph A, Diamond S, Butterfield CN, Thomas BC, Banfield JF. Novel soil bacteria possess diverse genes for secondary metabolite biosynthesis. Nature. 2018;558(7710):440-4.
12. Keren R, Mayzel B, Lavy A, Polishchuk I, Levy D, Fakra SC, et al. Sponge-associated bacteria mineralize arsenic and barium on intracellular vesicles. Nat Commun. 2017;8:14393.
13. Bewley CA, Holland ND, Faulkner DJ. Two classes of metabolites from *Theonella swinhoei* are localized in distinct populations of bacterial symbionts. Experientia. 1996;52(7):716-22.
14. Schmidt EW, Obraztsova AY, Davidson SK, Faulkner DJ, Haygood MG. Identification of the antifungal peptide-containing symbiont of the marine sponge *Theonella swinhoei* as a novel δ-proteobacterium, “Candidatus Entotheonella palauensis”. Mar Biol. 2000;136(6):969-77.
15. Wilson MC, Mori T, Rückert C, Uria AR, Helf MJ, Takada K, et al. An environmental bacterial taxon with a large and distinct metabolic repertoire. Nature. 2014;506(7486):58-62.
16. Mori T, Cahn JKB, Wilson MC, Meoded RA, Wiebach V, Martinez AFC, et al. Single-bacterial genomics validates rich and varied specialized metabolism of uncultivated *Entotheonella* sponge symbionts. Proc Natl Acad Sci U S A. 2018, 115(8):1718-23.
17. Lackner G, Peters EE, Helfrich EJ, Piel J. Insights into the lifestyle of uncultured bacterial natural product factories associated with marine sponges. Proc Natl Acad Sci. 2017;114(3):E347-E56.
18. Ueoka R, Uria AR, Reiter S, Mori T, Karbaum P, Peters EE, et al. Metabolic and evolutionary origin of actin-binding polyketides from diverse organisms. Nat Chem Biol. 2015;11(9):705-12.
19. Wakimoto T, Egami Y, Nakashima Y, Wakimoto Y, Mori T, Awakawa T, et al. Calyculin biogenesis from a pyrophosphate protoxin produced by a sponge symbiont. Nat Chem Biol. 2014;10(8):648-55.
20. Pawlik JR. The Chemical Ecology of Sponges on Caribbean Reefs: Natural Products Shape Natural Systems. BioScience. 2011;61(11):888-98.
21. Hentschel U, Hopke J, Horn M, Friedrich AB, Wagner M, Hacker J, et al. Molecular evidence for a uniform microbial community in sponges from different oceans. Appl. Environ. Microbiol. 2002;68(9):4431-40.
22. Lafi FF, Fuerst JA, Fieseler L, Engels C, Goh WWLG, Hentschel U. Widespread Distribution of Poribacteria in Demospongiae. Appl. Environ. Microbiol. 2009;75(17): 5695-9.
23. Taylor MW, Radax R, Steger D, Wagner M. Sponge-Associated Microorganisms: Evolution, Ecology, and Biotechnological Potential. Microbiol. Mol. Biol. Rev. 2007;71(2):295-347.
24. Kogawa M., Miyaoka R, Hemmerling F, Ando M, Yura K, Ide K, et al. Single-cell metabolite detection and genomics reveals uncultivated talented producer. PNAS Nexus. 2022;1(1):pgab007.
25. Kimura M, Wakimoto T, Egami Y, Tan KC, Ise Y, Abe I. Calyxamides A and B, cytotoxic cyclic peptides from the marine sponge *Discodermia calyx*. J Nat Prod. 2012;75(2):290-4.
26. Nakashima Y, Egami Y, Kimura M, Wakimoto T, Abe I. Metagenomic Analysis of the Sponge *Discodermia* Reveals the Production of the Cyanobacterial Natural Product Kasumigamide by ‘Entotheonella’. PLoS one. 2016;11(10):e0164468.
27. Helf MJ, Jud A, Piel J. Enzyme from an Uncultivated Sponge Bacterium Catalyzes S‐Methylation in a Ribosomal Peptide. ChemBioChem. 2017;18(5):444-50.
28. Reiter S, Cahn JK, Wiebach V, Ueoka R, Piel J. Characterization of an Orphan Type III Polyketide Synthase Conserved in Uncultivated “Entotheonella” Sponge Symbionts. ChemBioChem. 2020;21(4):564.
29. Jaspars M, Challis G. Microbiology: A talented genus. Nature. 2014;506(7486):38-9.
30. Miller IJ, Lopera JG, Montgomery K, Puglisi M, Rose W, Kwan JC. Deconvolution of complete microbial genomes from shotgun metagenomes. Planta Med. 2016;82(S 01):SL4.
31. Feng G, Sun W, Zhang F, Orlić S, Li Z. Functional Transcripts Indicate Phylogenetically Diverse Active Ammonia-Scavenging Microbiota in Sympatric Sponges. Mar Biotechnol. 2018;20(2):131-43.
32. Tong A-Z, Liu W, Liu Q, Xia G-Q, Zhu J-Y. Diversity and composition of the *Panax ginseng* rhizosphere microbiome in various cultivation modes and ages. BMC Microbiology. 2021;21(1):18.
33. Robbins S, Song W, Engelberts J, Glasl B, Slaby BM, Boyd J, et al. A genomic view of the microbiome of coral reef demosponges. ISME J. 2021;15(6):1641-54.
34. Robinson SJ, Tenney K, Yee DF, Martinez L, Media JE, Valeriote FA, et al. Probing the bioactive constituents from chemotypes of the sponge *Psammocinia* aff. *bulbosa*. J Nat Prod. 2007;70(6):1002-9.
35. Cárdenas P, Rapp HT. Disrupted spiculogenesis in deep-water Geodiidae (Porifera, Demospongiae) growing in shallow waters. Invertebr Biol. 2013;132(3):173-94.
36. Cárdenas P, Rapp HT, Klitgaard AB, Best M, Thollesson M, Tendal OS. Taxonomy, biogeography and DNA barcodes of *Geodia* species (Porifera, Demospongiae, Tetractinellida) in the Atlantic boreo-arctic region. Zool J Linn Soc. 2013;169(2):251-311.
37. Steffen K., Indraningrat A.A.G., Erngren I. et al. Oceanographic setting influences the prokaryotic community and metabolome in deep-sea sponges. Sci Rep.2022;12:3356.
38. Roume H, Heintz-Buschart A, Muller EEL, Wilmes P. Chapter Eleven - Sequential Isolation of Metabolites, RNA, DNA, and Proteins from the Same Unique Sample. In: DeLong EF, editor. Methods in Enzymology. 531: Academic Press; 2013. p. 219-36.
39. Edgar RC. MUSCLE: multiple sequence alignment with high accuracy and high throughput. Nucl Acids Res. 2004;32(5):1792-7.
40. Nei M, Kumar S. Molecular evolution and phylogenetics: Oxford University Press; 2000.
41. Felsenstein J. Confidence limits on phylogenies: an approach using the bootstrap. Evolution. 1985;39(4):783-91.
42. Tamura K, Stecher G, Peterson D, Filipski A, Kumar S. MEGA6: molecular evolutionary genetics analysis version 6.0. Mol Biol Evol. 2013;30(12):2725-9.
43. Letunic I, Bork P. Interactive tree of life (iTOL) v3: an online tool for the display and annotation of phylogenetic and other trees. Nucl Acids Res. 2016;44(W1):W242-W5.
44. Bushnell B. BBmap 2014 [Available from: sourceforge.net/projects/bbmap/].
45. Nurk S, Meleshko D, Korobeynikov A, Pevzner PA. metaSPAdes: a new versatile metagenomic assembler. Genome Res. 2017;27(5):824-34.
46. Uritskiy GV, DiRuggiero J, Taylor J. MetaWRAP—a flexible pipeline for genome-resolved metagenomic data analysis. Microbiome. 2018;6(1):158.
47. Chaumeil P-A, Mussig AJ, Hugenholtz P, Parks DH. GTDB-Tk: a toolkit to classify genomes with the Genome Taxonomy Database. Bioinformatics. 2019;36(6):1925-7.
48. Eren AM, Esen ÖC, Quince C, Vineis JH, Morrison HG, Sogin ML, et al. Anvi’o: an advanced analysis and visualization platform for ‘omics data. PeerJ. 2015;3:e1319.
49. Hyatt D, Chen G-L, LoCascio PF, Land ML, Larimer FW, Hauser LJ. Prodigal: prokaryotic gene recognition and translation initiation site identification. BMC Bionform. 2010;11(1):119.
50. Lee MD. GToTree: a user-friendly workflow for phylogenomics. Bioinformatics. 2019;35(20):4162-4.
51. Price MN, Dehal PS, Arkin AP. FastTree: computing large minimum evolution trees with profiles instead of a distance matrix. Mol Biol Evol. 2009;26(7):1641-50.
52. Blin K, Shaw S, Steinke K, Villebro R, Ziemert N, Lee SY, et al. antiSMASH 5.0: updates to the secondary metabolite genome mining pipeline. Nucl Acids Res. 2019;47(W1):W81-W7.
53. Parks DH, Imelfort M, Skennerton CT, Hugenholtz P, Tyson GW. CheckM: assessing the quality of microbial genomes recovered from isolates, single cells, and metagenomes. Genome Res. 2015;25(7):1043-55.
54. Seemann T. Prokka: rapid prokaryotic genome annotation. Bioinformatics. 2014;30(14):2068-9.
55. Aramaki T, Blanc-Mathieu R, Endo H, Ohkubo K, Kanehisa M, Goto S, et al. KofamKOALA: KEGG Ortholog assignment based on profile HMM and adaptive score threshold. Bioinformatics. 2019;36(7):2251-2.
56. Kanehisa M, Sato Y, Morishima K. BlastKOALA and GhostKOALA: KEGG Tools for Functional Characterization of Genome and Metagenome Sequences. J Mol Biol. 2016;428(4):726-31.
57. Yarza P, Yilmaz P, Pruesse E, Glöckner FO, Ludwig W, Schleifer K-H, et al. Uniting the classification of cultured and uncultured bacteria and archaea using 16S rRNA gene sequences. Nat Rev Microbiol. 2014;12(9):635-45.
58. Montalvo NF, Hill RT. Sponge-associated bacteria are strictly maintained in two closely related but geographically distant sponge hosts. Appl Environ Microbiol. 2011;77(20):7207-16.
59. Glöckner V, Wehrl M, Moitinho-Silva L, Gernert C, Schupp P, Pawlik JR, et al. The HMA-LMA dichotomy revisited: an electron microscopical survey of 56 sponge species. Biol Bull. 2014;227(1):78-88.
60. Lavy A, Keren R, Yu K, Thomas BC, Alvarez‐Cohen L, Banfield JF, et al. A novel Chromatiales bacterium is a potential sulfide oxidizer in multiple orders of marine sponges. Environ Microbiol. 2018;20(2):800-14.
61. Simister R, Taylor MW, Rogers KM, Schupp PJ, Deines P. Temporal molecular and isotopic analysis of active bacterial communities in two New Zealand sponges. FEMS Microbiol Ecol. 2013;85(1):195-205.
62. Kimes NE, Johnson WR, Torralba M, Nelson KE, Weil E, Morris PJ. The *Montastraea faveolata* microbiome: ecological and temporal influences on a Caribbean reef‐building coral in decline. Environ Microbiol. 2013;15(7):2082-94.
63. Roberts EM, Bowers DG, Meyer HK, Samuelsen A, Rapp HT, Cárdenas P. Water masses constrain the distribution of deep-sea sponges in the North Atlantic Ocean and Nordic Seas. Mar Ecol Prog Ser. 2021;659:75-96.
64. Liu Y, Lai Q, Dong C, Sun F, Wang L, Li G, et al. Phylogenetic diversity of the *Bacillus pumilus* group and the marine ecotype revealed by multilocus sequence analysis. PLoS one. 2013;8(11):e80097.
65. Field K, Gordon D, Wright T, Rappé M, Urback E, Vergin K, et al. Diversity and depth-specific distribution of SAR11 cluster rRNA genes from marine planktonic bacteria. Appl Environ Microbiol. 1997;63(1):63-70.
66. Sunagawa S, Coelho LP, Chaffron S, Kultima JR, Labadie K, Salazar G, et al. Structure and function of the global ocean microbiome. Science. 2015;348(6237).
67. Blanquer A, Uriz MJ, Galand PE. Removing environmental sources of variation to gain insight on symbionts vs. transient microbes in high and low microbial abundance sponges. Environ Microbiol. 2013;15(11):3008-19.
68. Pettit GR, Xu J-P, Chapuis J-C, Pettit RK, Tackett LP, Doubek DL, et al. Antineoplastic Agents. 520. Isolation and Structure of Irciniastatins A and B from the Indo-Pacific Marine Sponge *Ircinia ramosa*. J Med Chem. 2004;47(5):1149-52.
69. Cichewicz RH, Valeriote FA, Crews P. Psymberin, a potent sponge-derived cytotoxin from *Psammocinia* distantly related to the pederin family. Org Lett. 2004;6(12):1951-4.
70. Bielitza M, Pietruszka J. The psymberin story—biological properties and approaches towards total and analogue syntheses. Angew Chem Int Ed. 2013;52(42):10960-85.
71. Helfrich EJN, Piel J. Biosynthesis of polyketides by *trans*-AT polyketide synthases. Nat Prod Rep. 2016;33(2):231-316.
72. Rust M, Helfrich EJ, Freeman MF, Nanudorn P, Field CM, Rückert C, et al. A multiproducer microbiome generates chemical diversity in the marine sponge *Mycale hentscheli*. Proc Natl Acad Sci U S A. 2020;117(17):9508-18.
73. Fisch KM, Gurgui C, Heycke N, van der Sar SA, Anderson SA, Webb VL, et al. Polyketide assembly lines of uncultivated sponge symbionts from structure-based gene targeting. Nat Chem Biol. 2009;5:494.
74. Kogawa M., Miyaoka R, Hemmerling F, Ando M, Yura K, Ide K, et al. Single-cell metabolite detection and genomics reveals uncultivated talented producer. PNAS Nexus. 2022;1(1):pgab007.
75. Della Salla G, Hochmuth T, Constantino V, Testa R, Gerwick W, Gerwick L, et al. Polyketide genes in the marine sponge *Plakortis simplex*: a new group of mono-modular type I polyketide synthases from sponge symbionts. Environ Microbiol Rep. 2013;5(6):809-18.
76. Kato S, Masuda S, Shibata A, Shirasu K, Ohkuma M. Insights into ecological roles of uncultivated bacteria in Katase hot spring sediment from long-read metagenomics. Front. Microbiol. 2022;13:1045931.
77. Taylor JA, Palladino G, Wemheuer B, Steinert G, Sipkema D, Williams TJ, et al. Phylogeny resolved, metabolism revealed: functional radiation within a widespread and divergent clade of sponge symbionts. ISME J. 2021;15(2):503-19.
78. Moitinho-Silva L, Díez-Vives C, Batani G, Esteves AIS, Jahn MT, Thomas T. Integrated metabolism in sponge–microbe symbiosis revealed by genome-centered metatranscriptomics. ISME J. 2017;11(7):1651-66.
79. Yang M, Nightingale PD, Beale R, Liss PS, Blomquist B, Fairall C. Atmospheric deposition of methanol over the Atlantic Ocean. Proc Natl Acad Sci. 2013;110(50):20034-9.
80. Mincer TJ, Aicher AC. Methanol Production by a Broad Phylogenetic Array of Marine Phytoplankton. PLoS one. 2016;11(3):e0150820.
81. Turekian VC, Macko SA, Keene WC. Concentrations, isotopic compositions, and sources of size-resolved, particulate organic carbon and oxalate in near-surface marine air at Bermuda during spring. Journal of Geophysical Research: Atmospheres. 2003;108:4157.
82. Cerrano C, Bavestrello G, Arillo A, Benatti U, Bonpadre S, Cattaneo-Vietti R, et al. Calcium oxalate production in the marine sponge *Chondrosia reniformis*. Mar Ecol Prog Ser. 1999;179:297-300.
83. Engelberts JP, Robbins SJ, de Goeij JM, Aranda M, Bell SC, Webster NS. Characterization of a sponge microbiome using an integrative genome-centric approach. ISME J. 2020;14(5):1100-10.
84. Pita L, Rix L, Slaby BM, Franke A, Hentschel U. The sponge holobiont in a changing ocean: from microbes to ecosystems. Microbiome. 2018;6(1):46.

**Figure legends:**

**Figure 1: Phylogeny and environmental distribution of the candidate phylum Tectomicrobia based on 16S rRNA gene sequences.** A) Phylogenetic tree inferred from 456 tectomicrobial sequences under the neighbor-joining criterion. Support of individual branches by bootstrap values above 50% is indicated at the respective nodes for the neighbor-joining method (left) and the maximum-likelihood method (right). Scale bar, 0.01 changes per nucleotide position. The number of sequences comprising a specific group is shown inside or next to the corresponding clade. The phylum *Nitrospinota* was used as an outgroup. The environmental distribution of sequences assigned to specific groups is shown as a pie chart next to the corresponding group. The subgroups 't1' and 't2', present in Clade 2, contained 10 and 11 sequences of mainly terrestrial origin respectively, but further data are needed to resolve their relationship with the rest of the Clade. B) Median sequence identities within (main diagonal) and between (off-diagonal) the genus-level clades described in this study. A more detailed version of this figure, showing higher and lower-level divisions, is provided in Fig. S5. C) Pie chart diagram illustrating the environmental distribution of 'Tectomicrobia' based on 456 16S rRNA gene sequences. A list of sequences generated in this study can be found in Table S3

**Figure 2: Phylogeny of *'*Thalassonella'**. Detailed view of 'Thalassonella subgroup for the tree shown in Figure 2. Bootstrap values above 50% are given for the neighbor-joining (left) and maximum-likelihood (right) methods. Sequences generated in this study are indicated with a black circle. Scale bar, 0.005 changes per nucleotide position.

**Figure 3: Phylogeny of *'*Entotheonella'.** Detailed view of *'*Entotheonella' for the tree shown in Figure 2. Bootstrap values above 50% are given for the neighbor-joining (left) and maximum-likelihood method (right). Sequences generated in this study are indicated with a black circle and 'Entotheonella' bacteria previously linked to the production of bioactive natural products are highlighted with a black asterisk. Sequences derived from soil samples are highlighted in red. Scale bar, 0.01 changes per nucleotide position

**Figure 4:** **CARD-FISH localization of '*Ca.* Entotheonella consociata' in *Psammocinia* sp..** Overlay of a bright-field image of a representative thin slice of *Psammocinia* sp. (A) with a fluorescent image obtained from CARD-FISH labeling of 'Entotheonella' (B). Scale bar: 20 µm.

**Figure 5: Analysis of putative 'Tectomicrobia' members from shotgun metagenomics.** (A) Reproduction of Figure 1A showing the placement of 15 putative 'Tectomicrobia' bins in red by alignment of recovered 16S sequences~~.~~ (B) Unrooted phylogenomic tree of putative 'Tectomicrobia' MAGs containing 16S sequences based on concatenation of conserved single-copy genes, including for reference three 'Entotheonella' draft genomes ('E. factor', NCBI ID AZHW01; 'E. gemina', AZHX01; 'E. serta', PPXO01) and three draft genomes from the adjacent bacterial phylum Nitrospinae (MHDL01, PCWG01, DCWK01). The MAGs Gb4_35 and Gb10_1 showed a genome completeness below 75% and were therefore not included in the analyses of Table S6. Gb4_35 had an inconclusive taxonomic affiliation based on its 16S rRNA gene but was assigned to the ‘Thalassonella’ sublittoral to upper bathyal *Geodia* group through phylogenomics.

**Figure 6: BGC analysis of 'Thalassonella' MAGs.** (A) Table of conserved BGCs in 18 'Thalassonella'-assigned MAGs, including MAGs assigned on the basis of the phylogenomic tree in Figure 1B. MAGs containing an ortholog of a cluster are indicated with a '+', and other, non-conserved clusters are shown in the final column. (B) Representative architectures for the four conserved BGCs as found in ‘Thalassonella’. Putative transport elements are shown in green and regulatory elements in yellow, with core biosynthetic machinery in orange and tailoring enzymes in blue. For the latter two, predicted functions are assigned based on homology. For multi-domain enzymes, the domain architecture is elaborated, with a black circle representing a thiolation domain. For a conservation analysis of these clusters, along with a comparison to the related ‘Entotheonella’ BGCs, see Figs. S10-13.
