# Supplementary material for: Distribution and diversity of ‘Tectomicrobia’, a deep-branching uncultivated bacterial lineage harboring rich producers of bioactive metabolites
Source: ISME Commun. 2023 May 29;3:50. doi: 10.1038/s43705-023-00259-z (PMC10227082; doi:10.1038/s43705-023-00259-z)
Supplement: Supplementary file 2 — Supplementary information [file 43705_2023_259_MOESM2_ESM.pdf]

Supplementary information

Distribution and diversity of 'Tectomicrobia', a deep-branching uncultivated bacterial lineage harboring rich producers of bioactive metabolites

Eike E. Peters[1*], Jackson K. B. Cahn[1*], Alessandro Lotti[1*], Asimenia Gavriilidou[2], Ursula A. E. Steffens[3], Catarina Loureiro[2], Michelle A Schorn[2], Paco Cárdenas[4], Nilani Vickneswaran[3], Phillip Crews[5], Detmer Sipkema[2], Jörn Piel[1]

[1] Institute of Microbiology, Eidgenössische Technische Hochschule (ETH) Zürich, Vladimir-Prelog-Weg 4, 8093 Zürich, Switzerland.

[2] Laboratory of Microbiology, Wageningen University and Research, 6708 WE Wageningen, The Netherlands.

[3] Kekule Institute of Organic Chemistry and Biochemistry, University of Bonn, Gerhard-Domagk-Strasse 1, 53121 Bonn, Germany.

[4] Pharmacognosy, Department of Pharmaceutical Biosciences, BioMedical Center, Uppsala University, Husargatan 3, 75124, Uppsala, Sweden.

[5] Department of Chemistry and Biochemistry, University of California at Santa Cruz, Santa Cruz, California, United States.

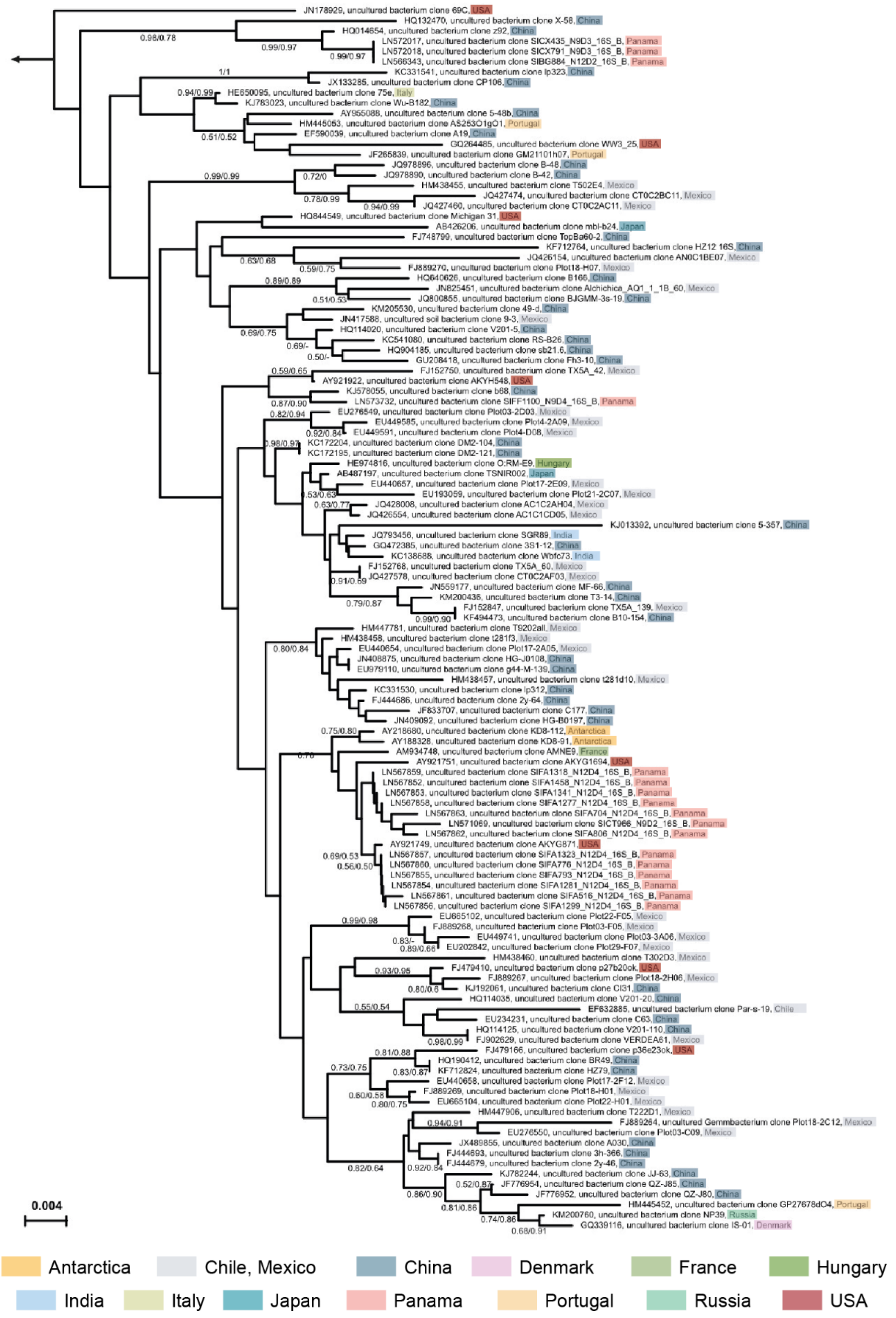

Supplementary Figure S1: Phylogeny of '*Ca.* Allonella'. Detailed view of '*Ca.* Allonella' genus of the tree shown in Figure 1. Bootstrap values above 50% are given for neighbor-joining (left) and maximum-likelihood (right). Scale bar, 0.004 changes per nucleotide position.

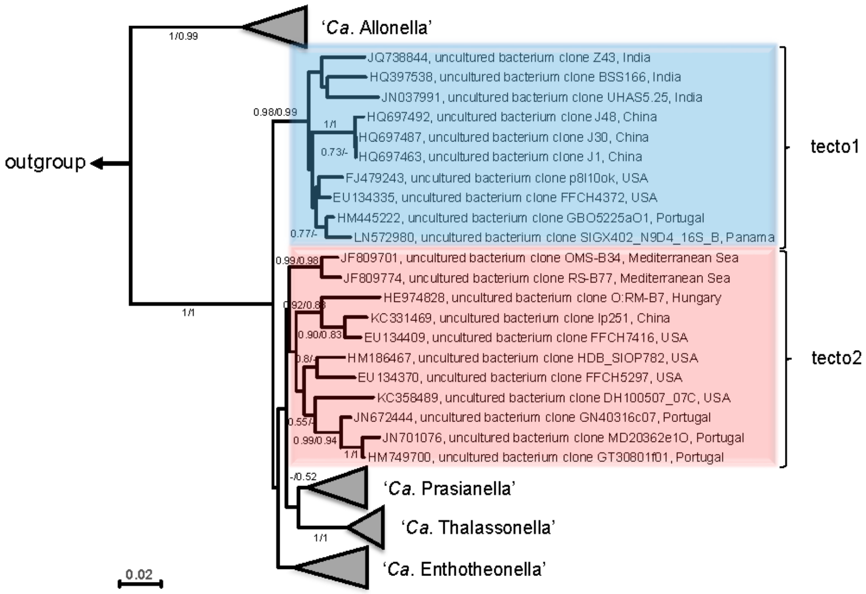

Supplementary Figure S2: Phylogeny of tecto1 and tecto2 subgroups. Detailed view of tecto1 and tecto2 subgroup of the tree shown in Figure 1. Bootstrap values above 50% are given for neighbor-joining (left) and maximum-likelihood (right). Scale bar, 0.02 changes per nucleotide position.

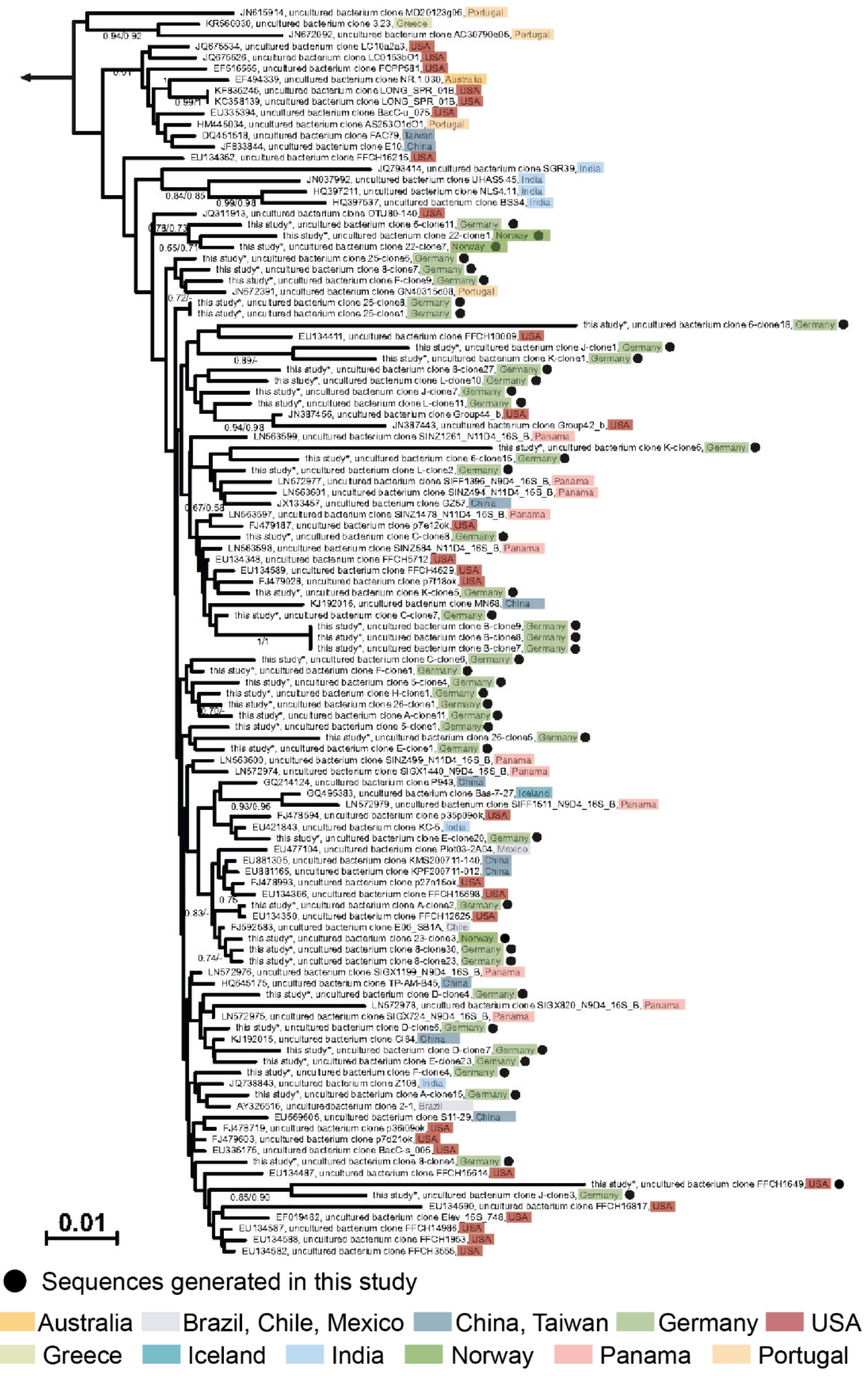

Supplementary Figure S3: Phylogeny of *'Ca.* Prasianella'. Detailed view of '*Ca*. Prasianella' genus of the tree shown in Figure 1. Bootstrap values above 50% are given for neighbor-joining (left) and maximum-likelihood (right). Sequences generated in this study are indicated with a black circle. The geographic origin for each sequence is highlighted in different colors as shown. Scale bar, 0.01 changes per nucleotide position.

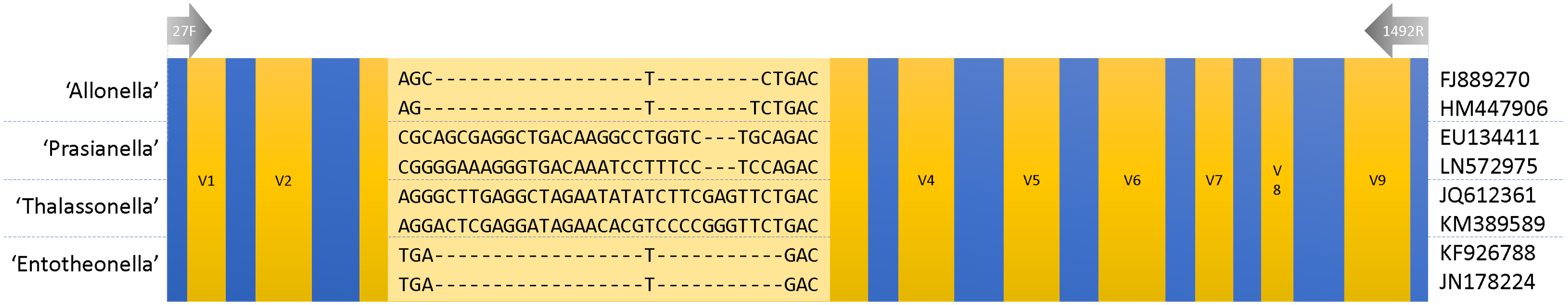

Supplementary Figure S4: 16S rRNA insertion in V3. Schematic of the 16S rRNA gene as sequenced from the 27F and 1492R primers, highlighting a 25-29 bp insertion in variable region 3. Variable regions are shown in gold and conserved regions in blue, with two representative sequences shown for each named candidate genus in 'Tectomicrobia'.

	‘Allonella’	Entotheonellaceae	‘Prasinella’	‘Thalassonella’	‘Entotheonella’	t1	t2	Upper bathyal Xestospongia ‘Thalassonella’	Sublittoral Geodia ‘Thalassonella’	Upper bathyal Geodia ‘Thalassonella’	Lower bathyal Geodia ‘Thalassonella’	‘Entotheonella’ group I	‘Entotheonella’ group IIa	‘Entotheonella’ group IIb	‘Entotheonella’ group IIc
‘Allonella’	94.3	80.8	80.4	79.9	81.9	80.1	80.1	80.0	79.8	79.8	80.1	82.2	82.0	82.5	81.7
Entotheonellaceae	80.8	88.0	89.0	88.7	87.3	86.9	88.0	88.7	88.8	88.6	89.1	87.1	87.0	88.0	87.0
‘Prasinella’	80.4	89.0	95.3	90.3	86.6	88.5	89.2	90.4	90.3	90.0	90.6	86.6	86.5	87.4	86.5
‘Thalassonella’	79.9	88.7	90.3	96.6	85.9	88.7	88.9	96.4	96.1	97.0	97.2	85.6	85.5	86.3	86.1
‘Entotheonella’	81.9	87.3	86.6	85.9	94.7	85.4	85.9	86.0	86.0	85.7	86.2	95.4	93.9	95.9	95.6
t1	80.1	86.9	88.5	88.7	85.4	92.5	89.1	88.9	88.8	88.4	89.0	85.4	85.6	85.7	85.1
t2	80.1	88.0	89.2	88.9	85.9	89.1	90.5	88.8	88.8	89.0	89.2	85.6	85.9	86.0	85.7
Upper bathyal Xestospongia ‘Thalassonella’	80.0	88.7	90.4	96.4	86.0	88.9	88.8	97.7	95.4	96.5	97.2	85.8	85.5	86.5	86.2
Sublittoral Geodia ‘Thalassonella’	79.8	88.8	90.3	96.1	86.0	88.8	88.8	95.4	98.2	95.8	95.7	85.8	85.7	86.4	86.1
Upper bathyal Geodia ‘Thalassonella’	79.8	88.6	90.0	97.0	85.7	88.4	89.0	96.5	95.8	98.4	97.3	85.5	85.4	86.0	85.9
Lower bathyal Geodia ‘Thalassonella’	80.1	89.1	90.6	97.2	86.2	89.0	89.2	97.2	95.7	97.3	99.0	85.8	85.7	86.7	86.4
‘Entotheonella’ group I	82.2	87.1	86.6	85.6	95.4	85.4	85.6	85.8	85.8	85.5	85.8	96.3	93.8	96.1	95.5
‘Entotheonella’ group IIa	82.0	87.0	86.5	85.5	93.9	85.6	85.9	85.5	85.7	85.4	85.7	93.8	98.5	93.6	93.2
‘Entotheonella’ group IIb	82.5	88.0	87.4	86.3	95.9	85.7	86.0	86.5	86.4	86.0	86.7	96.1	93.6	99.4	96.5
‘Entotheonella’ group IIc	81.7	87.0	86.5	86.1	95.6	85.1	85.7	86.2	86.1	85.9	86.4	95.5	93.2	96.5	97.3

Supplementary Figure S5: Detailed MSI heatmap. Median sequence identities within (main diagonal) and between (off-diagonal) the clades described in this study.

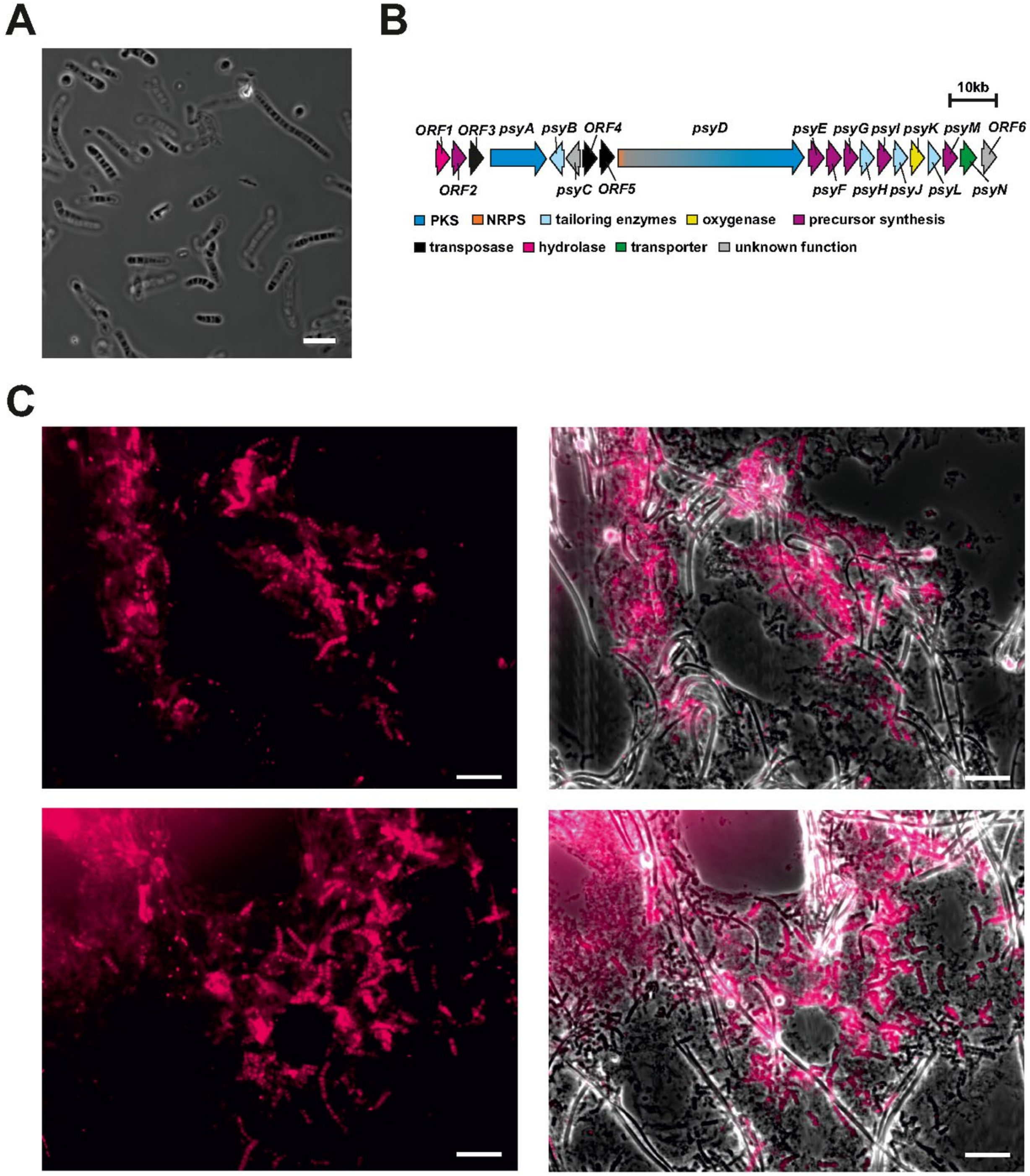

Supplementary Figure S6: '*Ca.* Entotheonella consociata' in *Psammocinia* sp. Phase contrast image of dissociated and for filamentous cells enriched cell pellet prepared from *Psammocina* sp. mainly showing '*Ca*. Entotheonella consociata'. Scale bar 5 µm. (B) Genomic organization of the *psy* biosynthetic gene cluster. (C) Overlay of a bright-field image of a representative thin slice of *Psammocinia* sp. (right) with a fluorescent image obtained from CARD-FISH labeling of 'Entotheonella' (left). Scale bar: 20 µm

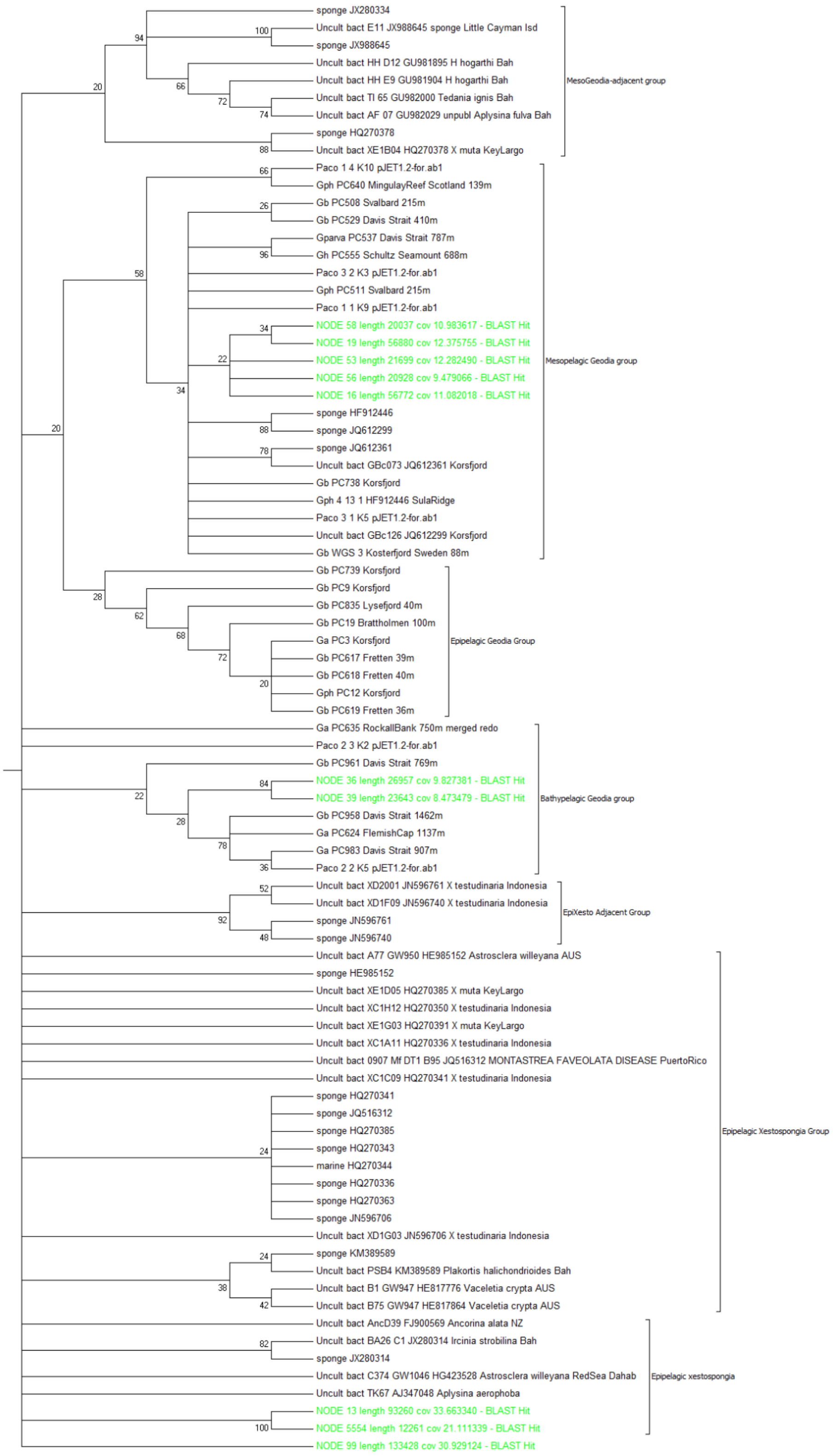

Supplementary Figure S7: Phylogeny of '*Ca.* Thalassonella' including MAGs. Maximum likelihood 16S rRNA phylogenetic tree of 'Thalassonella' reconstructed with the sequences of 10 MAGs (green). The tree is condensed below a 20% bootstrap value.

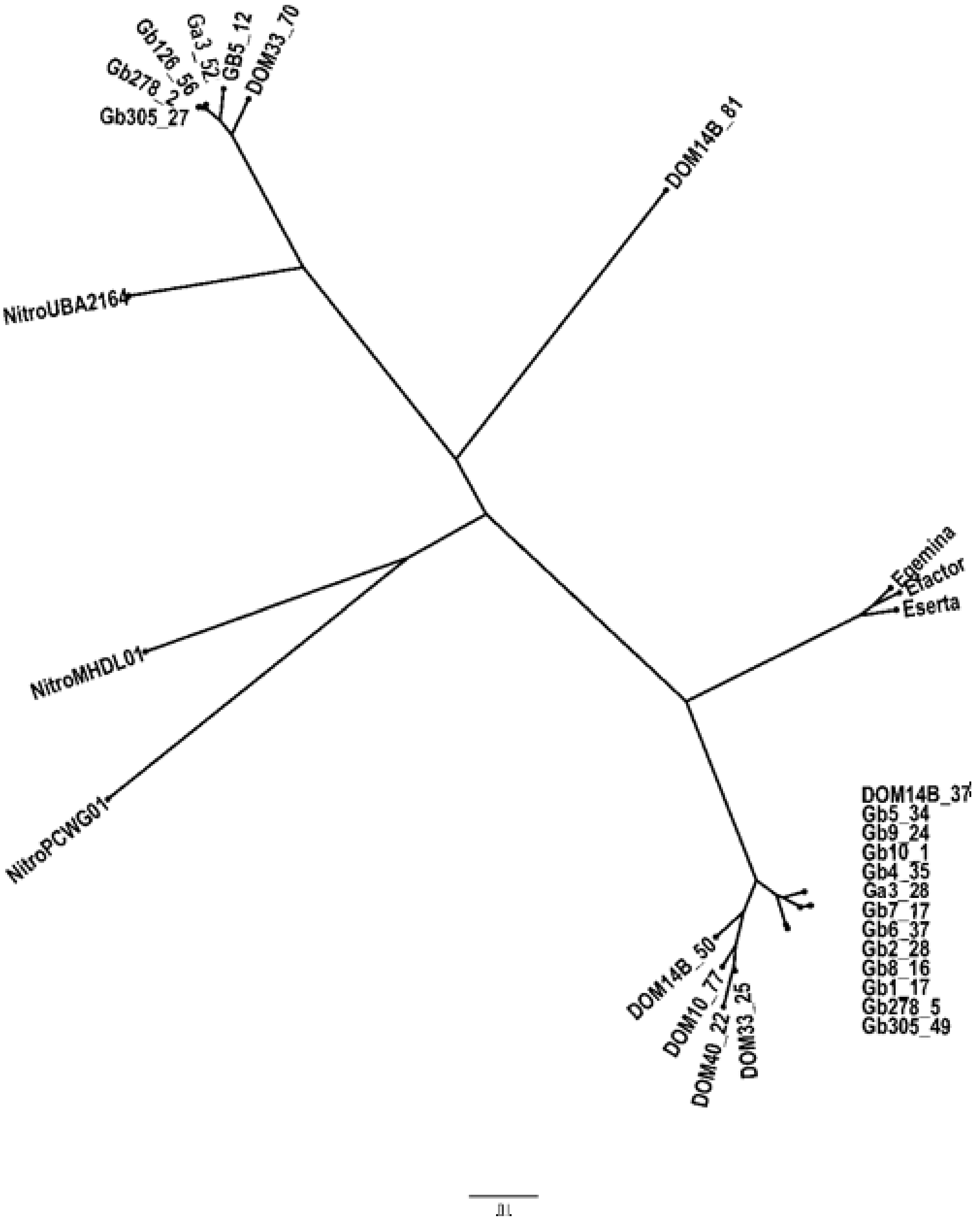

Supplementary Figure S8: Expanded phylogenomic tree of 'Tectomicrobia'. Unrooted Jukes-Cantor phylogenomic tree of putative 'Tectomicrobia' MAGs, including those without recovered 16S rRNA gene sequences, plus for reference three 'Entotheonella' draft genomes ('E. factor', NCBI ID AZHW01; 'E. gemina', AZHX01; 'E. serta', PPXO01) and three draft genomes from the adjacent bacterial phylum Nitrospinae (MHDL01, PCWG01, DCWK01).

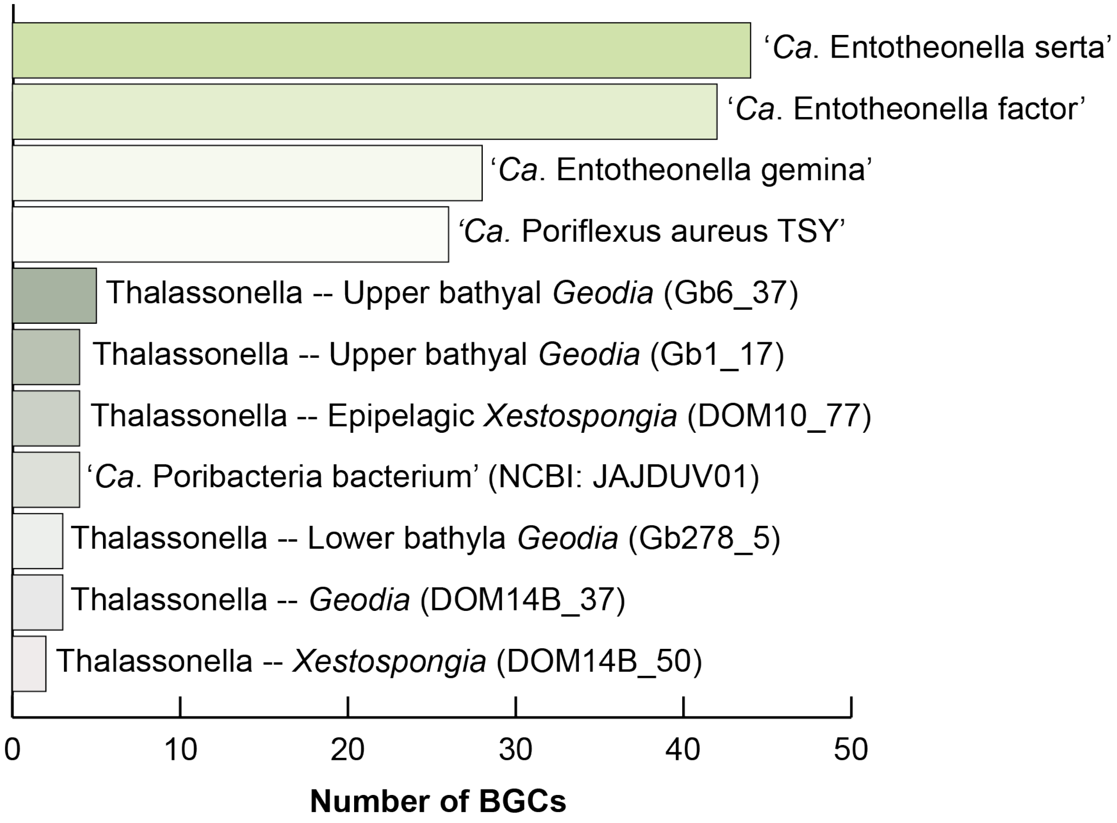

Supplementary Figure S9: Biosynthetic potential comparison between 'Thalassonella' 'Entotheonella' and other sponge symbionts. The graph shows the total number of biosynthetic gene clusters detected by antiSMASH 5.0 for three different 'Entotheonella' genomes, as well as *Chloroflexi* bacterium TSY, '*Ca*. Poribacteria bacterium' and six different 'Thalassonella' MAGs [1]. Highlighting the strong difference in biosynthetic potential between members of the 'Entotheonella' taxon compared to the 'Thalassonella' ones.

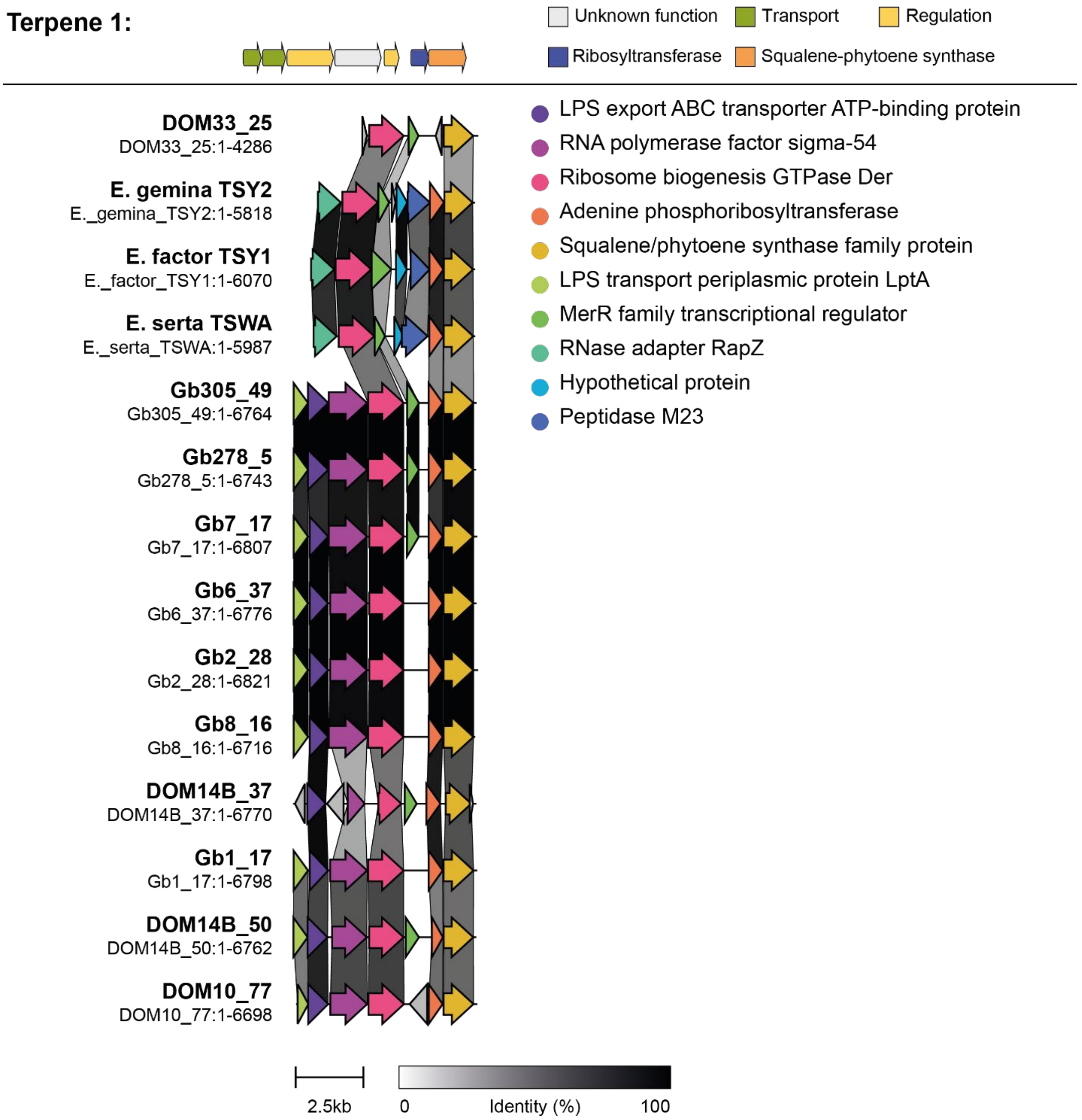

Supplementary Figure S10: Comparison of the putative 'terpene 1' biosynthetic gene clusters within 'Thalassonella' MAGs. The detected 'terpene 1' BGCs present in the available candidate 'Tectomicrobia' genomes were aligned and visualized using clinker v0.0.25 [2] followed by manual readjustment of the figure. A representative cluster used in Fig. 6B is shown at the top as reference. The genes were annotated based on homology using BLAST.

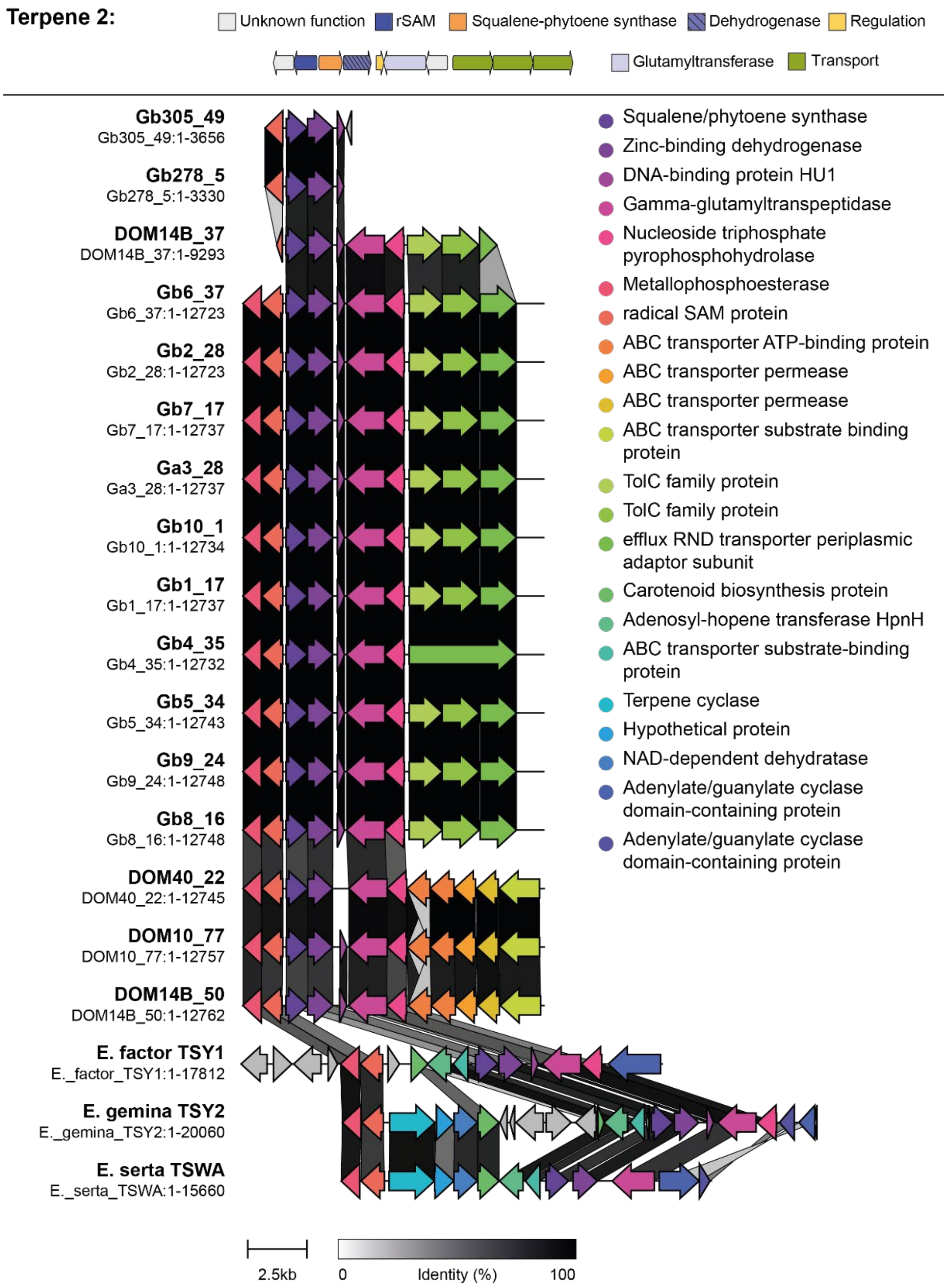

Supplementary Figure S11: Comparison of the putative 'terpene 2' biosynthetic gene clusters within 'Thalassonella' MAGs. All the detected 'terpene 2' BGCs present in the available candidate 'Tectomicrobia' genomes were aligned and visualized using clinker v0.0.25 [2] followed by manual readjustment of the figure. A representative cluster used in Fig. 6B is shown at the top as reference. The genes were annotated based on homology using BLAST.

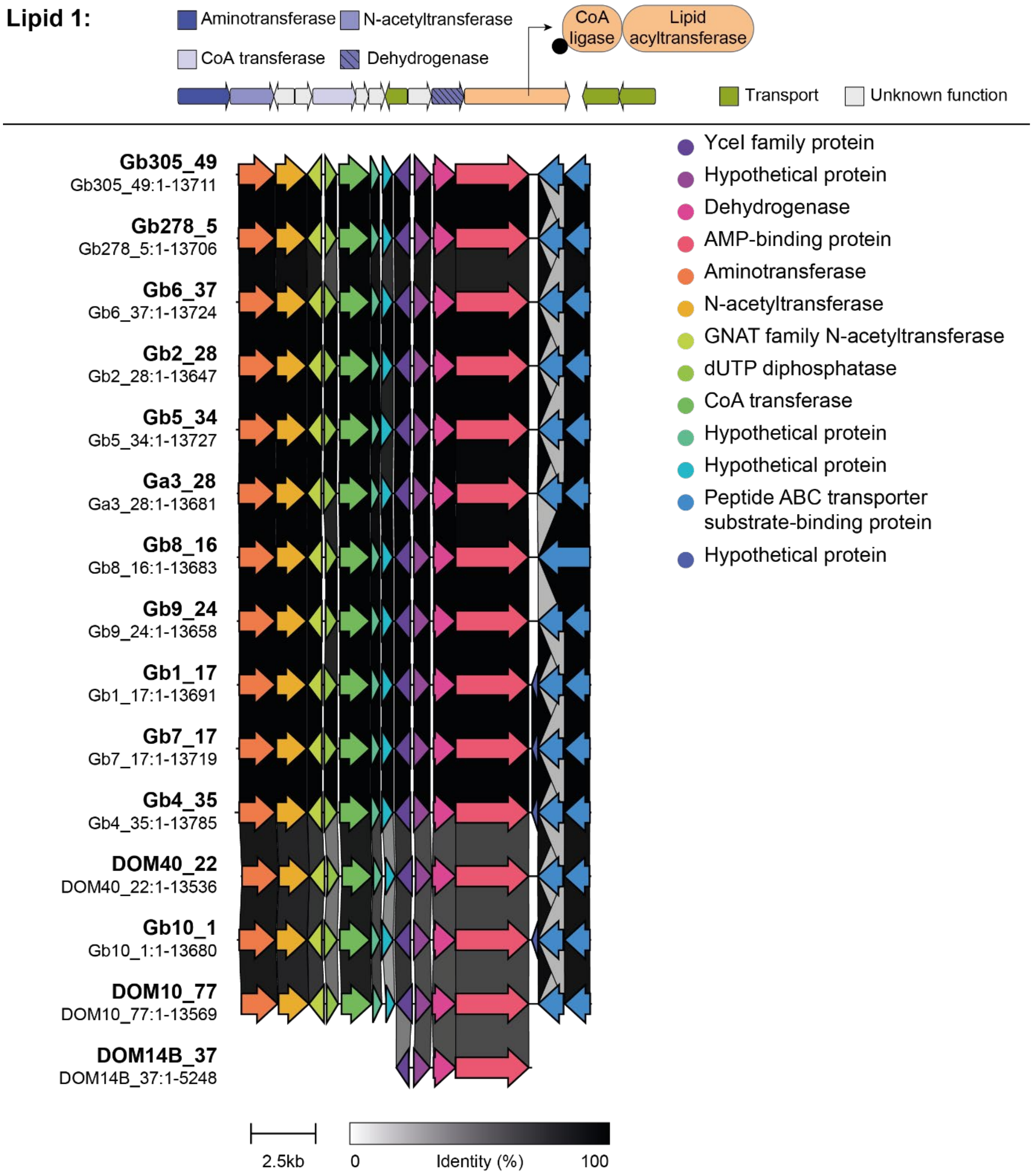

Supplementary Figure S12: Comparison of the putative 'lipid 1' biosynthetic gene clusters within 'Thalassonella' MAGs. The detected 'lipid 1' BGCs present in the available candidate 'Tectomicrobia' genomes were aligned and visualized using clinker v0.0.25 [2] followed by manual readjustment of the figure. A representative cluster used in Fig. 6B is shown at the top as reference. The genes were annotated based on homology using BLAST.

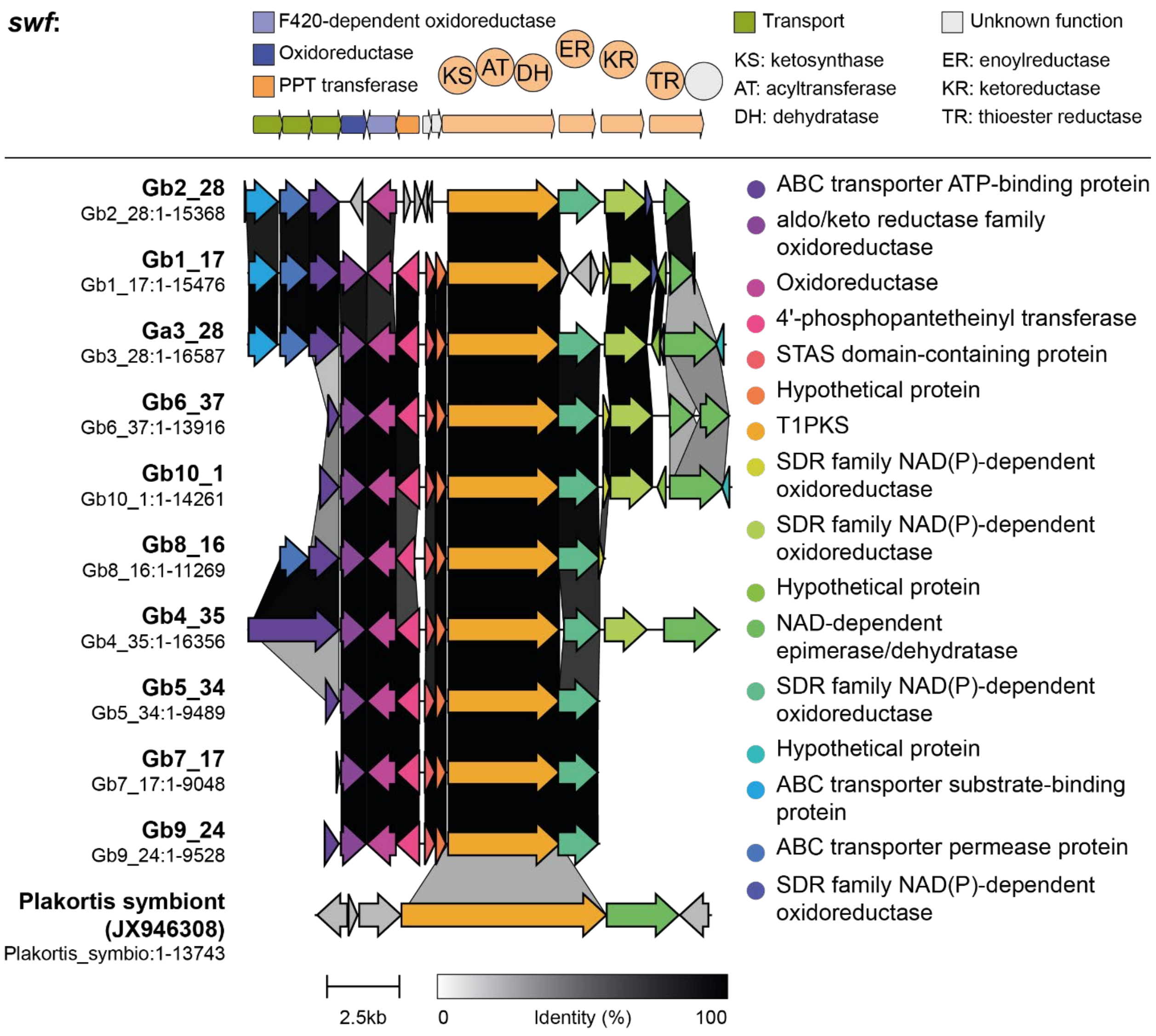

Supplementary Figure S13: Comparison of the putative '*swf*' biosynthetic gene clusters within 'Thalassonella' MAGs. All of the detected '*swf*' BGCs present in the available candidate 'Tectomicrobia' genomes were aligned and visualized using clinker v0.0.25 [2] followed by manual readjustment of the figure. A representative cluster used in Fig. 6B is shown at the top as reference. The genes were annotated based on homology using BLAST.

Supplementary Table S1. Metadata for sponge metagenomes that were used to reconstruct Tectomicrobia composite genomes

Sponge Host	Location	Sampling Year	Code	Bin (ENA sample accession #)	CheckM Complete. / Contam. (%)	16S length	16S ID	Anvi'o ID
Fascaplysinopsis sp.	Portsmouth, Dominica (N15°34'33.24", W61°27'20.16", 101m)	2016	DOM10	DOM10_77 (ERS12129423)	77.58 / 0.00	529	Thalassonella -- Sublittoral *Xestospongia*	Thalassonella -- Sublittoral *Xestospongia*
Geodia sp.	Portsmouth, Dominica (N15°34'33.24", W61°27'20.16", 230m)	2016	DOM14B	DOM14B_37 (ERS12129424)	63.79 / 0.00			Thalassonella -- Sublittoral to upper bathyal*Geodia*
				DOM14B_50 (ERS12129425)	92.3 / 1.71			Thalassonella -- Sublittoral *Xestospongia*
				DOM14B_81 (ERS12129426)	82.19 / 1.82			Outgroup (other)
Aplysina sp.	Cottage, Dominica (N15°36'54.66", W61°27'48.93", 106m)	2016	DOM33	DOM33_25 (ERS12129427)	76.96 / 0.85	483	Thalassonella -- Sublittoral *Xestospongia*	Thalassonella -- Sublittoral *Xestospongia*
				DOM33_70 (ERS12129428)	79.78 / 1.86	496	Outgroup Cluster 1	Outgroup Cluster 1
Desmacella sp.	Connor Bay, Dominica (N15°38'14.13", W61°27'39.13", 146m)	2016	DOM40	DOM40_22 (ERS12129429)	57.75 / 2.59			Thalassonella -- Sublittoral *Xestospongia*
Geodia barretti	Scengsbukt-Korsfjord, Norway (N60°8'8", E5°6'42", 450m)	2017	Gb1	Gb1_17 (ERS12129432)	92.83 / 1.71	627	Thalassonella -- Sublittoral to upper bathyal *Geodia*	Thalassonella – Sublittoral to upper bathyal *Geodia*
Geodia barretti	Scengsbukt-Korsfjord, Norway (N60°8'8", E5°6'42", 150m)	2017	Gb2	Gb2_28 (ERS12129433)	91.54 / 1.71	699	Thalassonella -- Sublittoral to upper bathyal *Geodia*	Thalassonella -- Sublittoral to upper bathyal *Geodia*
Geodia atlantica	Scengsbukt-Korsfjord, Norway (N60°8'8", E5°6'42", 150m)	2017	Ga3	Ga3_28 (ERS12129430)	65.51 / 0.00			Thalassonella -- Sublittoral to upper bathyal *Geodia*
				Ga3_52 (ERS12129431)	59.85 / 0.85	399	Outgroup cluster 1	Outgroup cluster 1
Geodia barretti	Scengsbukt-Korsfjord, Norway (N60°8'8", E5°6'42", 150m)	2017	Gb4	Gb4_35 (ERS12329860)	63.79 / 0.00	574	Tectomicrobia (singleton, unknown further taxonomy)	Thalassonella -- Sublittoral to upper bathyal *Geodia*
Geodia barretti	Scengsbukt-Korsfjord, Norway (N60°8'8", E5°6'42", 150m)	2017	Gb5	Gb5_34 (ERS12129434)	67.24 / 0.00			Thalassonella -- Sublittoral to upper bathyal *Geodia*
				Gb5_12 (ERS12129435)	75.96 / 0.85	460	Outgroup cluster 1	Outgroup cluster 1
Geodia barretti	Scengsbukt-Korsfjord, Norway (N60°8'8", E5°6'42", 150m)	2017	Gb6	Gb6_37 (ERS12129436)	91.97 / 2.56	747	Thalassonella -- Sublittoral to upper bathyal *Geodia*	Thalassonella -- Sublittoral to upper bathyal *Geodia*
Geodia barretti	Scengsbukt-Korsfjord, Norway (N60°8'8", E5°6'42", 450m)	2017	Gb7	Gb7_17 (ERS12129437)	92.02 / 1.71			Thalassonella -- Sublittoral to upper bathyal *Geodia*
Geodia barretti	Scengsbukt-Korsfjord, Norway (N60°8'8", E5°6'42", 450m)	2017	Gb8	Gb8_16 (ERS12129438)	91.97 / 2.56	613	Thalassonella -- Sublittoral to upper bathyal *Geodia*	Thalassonella -- Sublittoral to upper bathyal *Geodia*
Geodia barretti	Scengsbukt-Korsfjord, Norway (N60°8'8", E5°6'42", 450m)	2017	Gb9	Gb9_24 (ERS12129439)	65.51 / 0.00			Thalassonella -- Sublittoral to upper bathyal *Geodia*
Geodia barretti	Scengsbukt-Korsfjord, Norway (N60°8'8", E5°6'42", 450m)	2017	Gb10	Gb10_1 (ERS12129440)	65.51 / 0.00	496	Thalassonella -- Sublittoral to upper bathyal *Geodia*	Thalassonella -- Sublittoral to upper bathyal *Geodia*
Geodia barretti	Davis Strait, Canada (N62°52'15", W58°37'34", 1213m)	2015	Gb126	Gb126_56 (ERS12129441)	68.75 / 0.00			Outgroup cluster 1
Geodia barretti	Davis Strait, Canada (N61°53'36", W60°7'57", 1335m)	2014	Gb278	Gb278_2 (ERS12129442)	67.46 / 0.85	496	Outgroup cluster 1	Outgroup cluster 1
				Gb278_5 (ERS12129443)	92.27 / 4.27	633	Thalassonella -- Lower bathyal *Geodia*	Thalassonella – Lower bathyal *Geodia*
Geodia barretti	Davis Strait, Canada (N61°53'36", W60°7'57", 1437m)	2014	Gb305	Gb305_27 (ERS12129444)	67.62 / 0.00			Outgroup cluster 1
				Gb305_49 (ERS12129445)	80.17 / 1.72	638	Thalassonella -- Lower bathyal *Geodia*	Thalassonella -- Lower bathyal *Geodia*

Supplementary Table S2: List of soil samples used in this study. ND: Not determined

SAMPLE	*COLLECTION DATE*	*SOURCE*	*LOCATION*	*LATITUDE*	*LONGITUDE*
uncultured bacterium clone 22-clone1<br>*uncultured bacterium clone 22-clone7*	13.07.2012	soil	NO, Geilo	60.546011	8.24134
uncultured bacterium clone 23-clone3	14.07.2012	soil	NO, Drammen	ND	ND
uncultured bacterium clone 25-clone1<br>*uncultured bacterium clone 25-clone6*<br>*uncultured bacterium clone 25-clone8*	24.07.2012	soil	DE, Meckenheim	50.629311	7.048771
uncultured bacterium clone 26_clone2<br>*uncultured bacterium clone 26-clone1*<br>*uncultured bacterium clone 26-clone5*	24.07.2012	soil	DE, Meckenheim	50.629152	7.048804
uncultured bacterium clone 5-clone1<br>*uncultured bacterium clone 5-clone4*	26.05.2012	soil	DE, Bausendorf	50.016165	6.99383
uncultured bacterium clone 6-clone11<br>*uncultured bacterium clone 6-clone15*<br>*uncultured bacterium clone 6-clone18*	03.06.2012	sediment	DE, Kalenborn	50.557967	7.000637
uncultured bacterium clone 8-clone23<br>*uncultured bacterium clone 8-clone27*<br>*uncultured bacterium clone 8-clone30*<br>*uncultured bacterium clone 8-clone4*<br>*uncultured bacterium clone 8-clone7*<br>*uncultured bacterium clone-808*<br>*uncultured bacterium clone-811*<br>*uncultured bacterium clone-822*	05.06.2012	soil	DE, Berlin	ND	ND
uncultured bacterium clone 22-clone1	13.07.2012	soil	NO, Geilo	60.546011	8.24134
uncultured bacterium clone A-clone11<br>*uncultured bacterium clone A-clone2*	08.09.2012	soil	DE, Neuwied	50.438631	7.486529
uncultured bacterium clone B-clone7<br>*uncultured bacterium clone B-clone8*<br>*uncultured bacterium clone B-clone9*	08.09.2012	soil	DE, Neuwied	50.43848	7.486581
uncultured bacterium clone C-clone6<br>*uncultured bacterium clone C-clone7*<br>*uncultured bacterium clone C-clone8*	08.09.2012	soil	DE, Neuwied	50.427134	7.473694
uncultured bacterium clone D-clone4<br>*uncultured bacterium clone D-clone6*<br>*uncultured bacterium clone D-clone7*	08.09.2012	soil	DE, Neuwied	50.427196	7.473873
uncultured bacterium clone E-clone1<br>*uncultured bacterium clone E-clone20*<br>*uncultured bacterium clone E-clone23*	08.09.2012	soil	DE, Neuwied	50.424134	7.475721
uncultured bacterium clone F-clone1<br>*uncultured bacterium clone F-clone4*<br>*uncultured bacterium clone F-clone9*	08.09.2012	soil	DE, Neuwied	50.424405	7.475612
uncultured bacterium clone H-clone1	09.09.2012	soil	DE, Neuwied	50.425845	7.457446
uncultured bacterium clone I-clone1	09.09.2012	soil	DE, Neuwied	50.426084	7.455733
uncultured bacterium clone J-clone1<br>*uncultured bacterium clone J-clone3*<br>*uncultured bacterium clone J-clone7*	09.09.2012	soil	DE, Neuwied	50.425897	7.461494
uncultured bacterium clone K-clone1<br>*uncultured bacterium clone K-clone5*<br>*uncultured bacterium clone K-clone6*	09.09.2012	soil	DE, Neuwied	50.42583	7.46404
uncultured bacterium clone L-clone10<br>*uncultured bacterium clone L-clone11*<br>*uncultured bacterium clone L-clone2*	09.09.2012	soil	DE, Neuwied	50.425824	7.461559

Supplementary Table S3: List of (partial) 16S rRNA gene sequences generated in this study.

NCBI ACCESSION	SAMPLE	SOURC E	HOST	ORIGIN
OL753589	uncultured bacterium clone A-clone15	soil		Germany
OL753601	uncultured bacterium clone F-clone4	soil		Germany
OL753595	uncultured bacterium clone D-clone6	soil		Germany
OL753598	uncultured bacterium clone E-clone20	soil		Germany
OL753583	uncultured bacterium clone 26-clone5	soil		Germany
OL753577	uncultured bacterium clone 22-clone7	soil		Norway
OL753568	uncultured bacterium clone 6-clone11	soil		Germany
OL753579	uncultured bacterium clone 25-clone6	soil		Germany
OL753635	uncultured bacterium clone K-clone6	soil		Germany
OL753602	uncultured bacterium clone F-clone9	soil		Germany
OL753575	uncultured bacterium clone 8-clone30	soil		Germany
OL753573	uncultured bacterium clone 8-clone23	soil		Germany
OL753582	uncultured bacterium clone 26-clone1	soil		Germany
OL753567	uncultured bacterium clone 5-clone4	soil		Germany
OL753633	uncultured bacterium clone J-clone7	soil		Germany
OL753634	uncultured bacterium clone K-clone5	soil		Germany
OL753587	uncultured bacterium clone A-clone2	soil		Germany
OL753592	uncultured bacterium clone C-clone7	soil		Germany
OL753570	uncultured bacterium clone 6-clone18	soil		Germany
OL753603	uncultured bacterium clone PC3 200-400m	sponge	*Geodia atlantica*	Norway
OL753613	uncultured bacterium PC618 40m	sponge	*Geodia barretti*	Norway
OL753612	uncultured bacterium PC617 39m	sponge	*Geodia barretti*	Norway
OL753614	uncultured bacterium clone PC619 36m	sponge	*Geodia barretti*	Norway
OL753608	uncultured bacterium PC19 100m	sponge	*Geodia barretti*	Norway
OL753619	uncultured bacterium PC835 40m	sponge	*Geodia barretti*	Norway
OL753606	uncultured bacterium clone PC9 200-400m	sponge	*Geodia barretti*	Norway
OL753618	uncultured bacterium PC739 200-400m	sponge	*Geodia barretti*	Norway
OL753625	uncultured bacterium clone PC577_K2 1172m	sponge	*Geodia pachydermata*	Spain
OL753621	uncultured bacterium clone PC958_K5 1462m	sponge	*Geodia barretti*	Davis Strait
OL753615	uncultured bacterium clone PC619_K10 36m	sponge	*Geodia barretti*	Norway
OL753628	uncultured bacterium PC537 787m	sponge	*Geodia parva*	Norway
OL753623	uncultured bacterium PC981 88m	sponge	*Geodia barretti*	Sweden
OL753639	uncultured Entotheonella sp. clone DK10	sponge	*Discodermia kiiensis*	Japan
OL753584	uncultured bacterium clone-808	soil		Germany
OL753585	uncultured bacterium clone-811	soil		Germany
OL753581	uncultured bacterium clone 26_clone2	soil		Germany
OL753586	uncultured bacterium clone-822	soil		Germany
OL753644	uncultured Entotheonella sp. clone TSY2	sponge	*Theonella swinhoei Y*	Japan
OL753631	uncultured bacterium clone I-clone1	sponge		Germany
OL753571	uncultured bacterium clone 8-clone4	soil		Germany
OL753599	uncultured bacterium clone E-clone23	soil		Germany
OL753596	uncultured bacterium clone D-clone7	soil		Germany
OL753594	uncultured bacterium clone D-clone4	soil		Germany
OL753580	uncultured bacterium clone 25-clone8	soil		Germany

OL753566	uncultured bacterium clone 5-clone1	soil		Germany
OL753576	uncultured bacterium clone 22-clone1	soil		Norway
OL753572	uncultured bacterium clone 8-clone7	soil		Germany
OL753597	uncultured bacterium clone E-clone1	soil		Germany
OL753630	uncultured bacterium clone H-clone1	soil		Germany
OL753588	uncultured bacterium clone A-clone11	soil		Germany
OL753591	uncultured bacterium clone C-clone6	soil		Germany
OL753578	uncultured bacterium clone 23-clone3	soil		Norway
OL753600	uncultured bacterium clone F-clone1	soil		Germany
OL753593	uncultured bacterium clone C-clone8	soil		Germany
OL753590	uncultured bacterium clone B-clone9	soil		Germany
OL753569	uncultured bacterium clone 6-clone15	soil		Germany
OL753636	uncultured bacterium clone L-clone2	soil		Germany
OL753638	uncultured bacterium clone L-clone11	soil		Germany
OL753574	uncultured bacterium clone 8-clone27	soil		Germany
OL753637	uncultured bacterium clone L-clone10	soil		Germany
OL753626	uncultured bacterium PC12 200-400m	sponge	*Geodia phlegraei*	Norway
OM256467	uncultured bacterium PC635 750m	sponge	*Geodia atlantica*	Rockall Bank
OL753605	uncultured bacterium PC983 907m	sponge	*Geodia atlantica*	Davis Strait
OL753620	uncultured bacterium clone PC958 1462m	sponge	*Geodia barretti*	Davis Strait
OL753604	uncultured bacterium PC624 1137m	sponge	*Geodia atlantica*	Flemish Cap
OL753622	uncultured bacterium clone PC961 769m	sponge	*Geodia barretti*	Davis Strait
OL753629	uncultured bacterium PC640 139m	sponge	*Geodia phlegraei*	Scotland
OL753624	uncultured bacterium clone PC555 688m	sponge	*Geodia hentscheli*	Norway
OL753616	uncultured bacterium clone PC738 200-400m	sponge	*Geodia barretti*	Norway
OL753617	uncultured bacterium clone PC738_K5 200-400m	sponge	*Geodia barretti*	Norway
OL753607	uncultured bacterium clone PC12_K9 200-400m	sponge	*Geodia phlegraei*	Norway
OL753627	uncultured bacterium PC511 215m	sponge	*Geodia phlegraei*	Svalbard
OL753611	uncultured bacterium PC529 410m	sponge	*Geodia barretti*	Davis Strait
OL753610	uncultured bacterium PC510 215m	sponge	*Geodia macandrewii*	Svalbard
OL753609	uncultured bacterium PC508 215m	sponge	*Geodia barretti*	Svalbard
OL753641	uncultured Entotheonella sp. clone TSW-IS1	sponge	*Theonella swinhoei W*	Israel
OL753632	uncultured bacterium clone I-clone7	soil		Germany
OL753642	uncultured Entotheonella sp. clone TSW-JA1	sponge	*Theonella swinhoei W*	Japan
OL753643	uncultured Entotheonella sp. clone TSW-JA2	sponge	*Theonella swinhoei W*	Japan
OL753640	uncultured Entotheonella sp. clone PsAB	sponge	*Psammocina* sp.	Papua New Guinea

Supplementary Table S4: ORFs detected on the loci containing the *psy* genes and their putative functions

ORF	PROPOSED FUNCTION	CLOSEST HOMOLOG (SOURCE ORGANISM)	% AA IDENTITY	ACESSION NUMBER
**ORF1**	Zn-dependent hydrolase	MBL fold metallo-hydrolase (*Candidatus* Entotheonella palauensis)	81	WP_089939010.1
**ORF2**	Adenylosuccinate synthetase	Adenylosuccinate synthase (*Candidatus* Entotheonella palauensis)	94	WP_089939008
**ORF3**	Transposase	IS4 family transposase (*Phormidesmis priestleyi*)	46	WP_073074803.1
**PSYA**	Trans-AT PKS	PedI (*Pederus fuscipes* symbiont)	41	AAR19304.1
**PSYB**	Methyltransferase	PedA (*Pederus fuscipes* symbiont)	52	AAS47557.1
**PSYC**	pedK-like	OnnF (*Candidatus* Entotheonella factor)	42	AAV97874.1
**ORF4**	Transposase	IS4 family transposase (*Phormidesmis priestleyi*)	46	WP_073074803.1
**ORF5**	Transposase	Hypotetical protein (*Fimbriiglobus ruber*)	43	WP_088252106.1
**PSYD**	PKS-NRPS	PedF (*Pederus fuscipies* symbiont)	44	AAS47564.1
**PSYE**	Phosphoenolpyruvate synthase β and γ subunit	Phosphoenolpyruvate synthase β subunit/ γ subunit (*Discodermia dissoluta* symbiont)	93/92	AAY00048.1/ AAY00046.1
**PSYF**	Phosphosterase	Phosphoesterase SA1_PKS A (*Discodermia dissoluta* symbiont)	96	AY907538
**PSYG**	Phosphoenolpyurvate synthase α subunit	Phosphoenolpyruvate synthase α subunit (*Discodermia dissoluta* symbiont)	95	AAY00044.1
**PSYH**	acyltransferase	Acyltransferase (*Discodermia dissoluta* symbiont)	93	AAY00043.1
**PSYI**	HMG-CoA-synthase	HMG-CoA-synthase (*Discodermia dissoluta* symbiont)	98	AAY00042.1
**PSYJ**	Crotonase superfamily	Crotonase superfamily (*Discodermia dissoluta* symbiont)	95	AAY00041.1
**PSYK**	Flavin-dependent oxygenase	Flavin dependent oxygenase (*Discodermia dissoluta* symbiont)	96	AAY00040.1
**PSYL**	ACP	Crotonase superfamily (*Discodermia dissoluta* symbiont)	99	AAY00039.1
**PSYM**	3-oxoacyl ACP synthase	3-oxoacyl ACP synthase (*Discodermia dissoluta* symbiont)	96	AAY00038.1
**PSYN**	Cation transport ATPase	Cation transport ATPase (*Discodermia dissoluta* symbiont)	95	AAY00037.1
**ORF6**	unknown	hypothetical protein (*Candidatus* Entotheonella factor)	61	ETW92637.1

Table S5: 'Thalassonella' MAGs statistics and isolation source information.

MAG	Source	#Contigs	Genome size (bp)	GC content (%)	N50 (bp)	Completeness (%)	Contamination (%)	#Genes
Gb1_17	*Geodia barretti*	206	3,799,105	62.0	39,894	92.8	1.7	3,680
Gb2_28	*Geodia barretti*	239	3,975,034	62.0	43,132	91.5	1.7	3,846
Gb6_37	*Geodia barretti*	238	3,978,631	61.9	46,811	92.0	2.6	3,887
Gb8_16	*Geodia barretti*	181	3,896,062	61.9	47,852	92.0	2.6	3,744
Gb278_5	*Geodia barretti*	167	3,628,785	62.2	81,152	92.3	4.3	3,494
Gb305_49	*Geodia barretti*	173	3,559,325	62.4	78,347	80.2	1.7	3,428
DOM10_77	*Fascaplysinopsis* sp.	380	3,220,299	65.8	112,146	77.6	0.0	3,178
DOM33_25	*Aplysina* sp.	300	2,731,884	66.6	14,938	77.0	0.9	2,611

For Table S6, see Supplementary File.

References:

1. Blin K, Shaw S, Steinke K, Villebro R, Ziemert N, Lee SY, et al. antiSMASH 5.0: updates to the secondary metabolite genome mining pipeline. Nucl Acids Res. 2019;47(W1):W81-W7.
2. Gilchrist CLM, Chooi YH. clinker & clustermap.js: automatic generation of gene cluster comparison figures. *Bioinformatics*. 2021; 37(16):2473-75.
